# A polyketide synthase from *Verticillium dahliae* modulates melanin biosynthesis and hyphal growth to promote virulence

**DOI:** 10.1186/s12915-022-01330-2

**Published:** 2022-05-30

**Authors:** Huan Li, Dan Wang, Dan-Dan Zhang, Qi Geng, Jun-Jiao Li, Ruo-Cheng Sheng, Hui-Shan Xue, He Zhu, Zhi-Qiang Kong, Xiao-Feng Dai, Steven J. Klosterman, Krishna V. Subbarao, Feng-Mao Chen, Jie-Yin Chen

**Affiliations:** 1 Co-Innovation Center for Sustainable Forestry in Southern China, Nanjing Forestry University, Nanjing, 210037 Jiangsu China; 2 State Key Laboratory for Biology of Plant Diseases and Insect Pests, Institute of Plant Protection, Chinese Academy of Agricultural Sciences, Beijing, 100193 China; 3 National Cotton Industry Technology System Liaohe Comprehensive Experimental Station, The Cotton Research Center of Liaoning Academy of Agricultural Sciences, Liaoning Provincial Institute of Economic Crops, Liaoyang, 111000 China; 4 United States Department of Agriculture, Agricultural Research Service, Salinas, CA USA; 5 Department of Plant Pathology, University of California, Davis, c/o United States Agricultural Research Station, Salinas, CA USA

**Keywords:** *Verticillium dahliae*, Polyketide synthase, Melanin, Microsclerotia, Hyphal growth, Virulence

## Abstract

**Background:**

During the disease cycle, plant pathogenic fungi exhibit a morphological transition between hyphal growth (the phase of active infection) and the production of long-term survival structures that remain dormant during “overwintering.” *Verticillium dahliae* is a major plant pathogen that produces heavily melanized microsclerotia (MS) that survive in the soil for 14 or more years. These MS are multicellular structures produced during the necrotrophic phase of the disease cycle. Polyketide synthases (PKSs) are responsible for catalyzing production of many secondary metabolites including melanin. While MS contribute to long-term survival, hyphal growth is key for infection and virulence, but the signaling mechanisms by which the pathogen maintains hyphal growth are unclear.

**Results:**

We analyzed the VdPKSs that contain at least one conserved domain potentially involved in secondary metabolism (SM), and screened the effect of *VdPKS* deletions in the virulent strain AT13. Among the five *VdPKSs* whose deletion affected virulence on cotton, we found that *VdPKS9* acted epistatically to the *VdPKS1*-associated melanin pathway to promote hyphal growth. The decreased hyphal growth in *VdPKS9* mutants was accompanied by the up-regulation of melanin biosynthesis and MS formation. Overexpression of *VdPKS9* transformed melanized hyphal-type (MH-type) into the albinistic hyaline hyphal-type (AH-type), and *VdPKS9* was upregulated in the AH-type population, which also exhibited higher virulence than the MH-type.

**Conclusions:**

We show that VdPKS9 is a powerful negative regulator of both melanin biosynthesis and MS formation in *V. dahliae*. These findings provide insight into the mechanism of how plant pathogens promote their virulence by the maintenance of vegetative hyphal growth during infection and colonization of plant hosts, and may provide novel targets for the control of melanin-producing filamentous fungi.

**Supplementary Information:**

The online version contains supplementary material available at 10.1186/s12915-022-01330-2.

## Background

SM in fungi is important for morphological development, niche adaptation, reproduction, and to overcome stress [1–3]. In phytopathogenic fungi, pigments and mycotoxins play important roles in their development and virulence. Examples include aurofusarin [4], T-toxin [5], and fusaric acid [6]. Fungal genes encoding PKSs and regulatory genes are often linked in clusters in their genomes and are responsible for catalyzing the production of many SMs [7–9].

Melanin is produced by a broad variety of pathogenic microorganisms including bacteria, fungi and helminths, and its production may be stimulated as a stress response [10–14]. DHN-melanin biosynthesis is a highly conserved pathway in fungi and is initiated by a PKS that catalyzes the conversion of acetyl-CoA to 1,3,6,8-tetrahydroxynaphthalene (1,3,6,8-THN) followed by a series of downstream reactions catalyzed by reductases and dehydratases that form 1,8-dihydroxynaphthalene (1,8-DHN), which finally is catalyzed by laccase into melanin macromolecules [15]. The DHN melanin biosynthesis pathway members such as *ALB1*, *RSY1*, *BUF1*, *Pig1*, and *4HNR* have been identified in *Magnaporthe oryzae* [16–19]. In *Botrytis cinerea*, *bcpks12* and *bcpks13* are responsible for the deposition of melanin in sclerotia and conidia [15, 20, 21], respectively. The functional role of melanin in fungi has also been well documented. In *M. oryzae* and *Colletotrichum gloeosporioides*, melanin is a prerequisite for the formation of functional appressoria that accumulate turgor pressure necessary to penetrate plant cell walls [22–24]. Melanized sclerotia of *Sclerotinia sclerotiorum* and *B. cinerea* are essential for vegetative growth, long-term survival, and pathogenicity [25–27]. In *Hortaea werneckii*, melanin is important to adapt to osmotic stress, maintenance of cell wall integrity, and normal cell division [28]. Similarity in *V. dahliae*, typical PKS gene clusters that mediate melanin synthesis have been reported. Mutation of either the transcription factor *VdCmr1*, or the *VdPKS1*, resulted in the production of transparent MS that also compromised the responses of *V. dahliae* to stress factors, but neither reduced its virulence [12, 29].

*Verticillium dahliae* is a soil-borne phytopathogenic fungus that causes Verticillium wilt on more than 200 species of dicots [30–32] including many agriculturally important crops and woody plants [33, 34]. Infections lead to yellowing and wilting of foliage, abscission, vascular tissue discoloration, and eventual death [35, 36]. Secondary metabolites in *V. dahliae* play important roles in its growth and adaptation to environmental stresses, and in pathogenicity [37–40]. Examples include *VdCYP1* [37] involved in the synthesis of the wilting compound sulfacetamide, and *VdDf6* [40], a PKS_ER domain containing gene transferred horizontally from *Fusarium oxysporum* [38] that mediates *N*-lauroylethanolamine (NAE)-induced defoliation via interference with the host fatty acid metabolism and hormone levels.

*V. dahliae* is difficult to control, owing in part to the production of heavily melanized MS that survive in the soil for 14 or more years [31, 41, 42]. The dormant MS develop as multicellular structures from the septate hyphae and are composed of a cluster of compressed, thick-walled, pigmented cells [43]. MS produced in senescing plant tissues are resistant to biotic and abiotic stresses [12, 44], and serve as the primary inoculum for subsequent crops [45, 46]. Because of the importance of MS in the disease cycle, a number of studies have examined the signal transduction pathways and other developmental genes that regulate the formation of MS and the link between their production and pathogenicity in *V. dahliae* [45, 47–54]. For instance, VMK1 was the first mitogen-activated protein kinase (MAPK) characterized as a positive regulator of MS development and pathogenicity [55]; *VdHog1*, a homolog of the yeast high-osmolarity glycerol (HOG) response kinase, is responsible for the development of melanized MS that are resistant to osmotic stress [56].

The widely accepted view is that the development of MS is accompanied by DHN-melanin biosynthesis and that melanin is extruded between the plasma membrane and cell wall, and is impregnated into the cell wall [44, 57–59]. Many genes upstream of the melanin biosynthesis pathway also influence its biosynthesis in *V. dahliae*. For example, DHN pathway member *Vayg1* catalyzes the transformation of melanin precursor heptaketide naphthopyrone (YWA1) to 1,3,6,8-THN [44]. The transmembrane proteins, VdMsb and VdSho1, interact to transmit signals to a MAPK pathway and have been associated with melanin and MS production [60–62]. Deletion of transcription factor Som1 upstream of the DHN melanin pathway [63] resulted in hyaline colonies and colorless MS. Even though melanin biosynthesis in *V. dahliae* is regulated by *VdCmr1* and *VdPKS1* found within an SM gene cluster, knockouts of both genes revealed that melanin biosynthesis can be independent of MS formation [12]. In addition, MS formation is a key morphological characteristic differentiating *V. dahliae* from many other species within the genus [64]. *V. dahliae* strains may be classified into those of the microsclerotial-type, hyphal-type, and the intermediate type, according to their colony morphology and melanin accumulation [65–67]. In a few cases reported, the hyphal-type exhibited increased virulence relative to the microsclerotial-type [68].

Fungal PKSs, belonging to the multi-module type I PKS, catalyze the production of the most abundant polyketide compounds and fatty acid derivatives [69]. The core modules within these PKSs include ketosynthase (KS), acyltransferase (AT), and acyl-carrier (ACP) domains that catalyze polyketide synthesis. The maturation of these molecules occurs following modifications catalyzed by dehydratase (DH), methyltransferase (MT), ketoreductase (KR), and enoylreductase (ER) [70, 71]. There are at least 25 potential SM clusters in the *V. dahliae* genome, implicated in a variety of processes, including biosynthesis of DHN-melanin, ferricrocin, triacetyl fusarinine, and fujikurin [72]. However, the pathways and SM production mediated by *V. dahliae* PKS genes and their contributions to pathogenicity and morphological characteristics have not been systematically analyzed.

In this study, PKS genes from *V. dahliae* genome were systematically analyzed and screened in pathogenicity analyses, demonstrating that *VdPKS9* is a crucial virulence factor. Further results demonstrate that the hypothetical oxidoreductase VdPKS9 strongly negatively regulates development of MS and melanin biosynthesis. Analyses of *VdPKS9* function in a *VdPKS1* (required in melanin biosynthesis) mutant background revealed that *VdPKS9* is dominant to that of *VdPKS1*, and thus regulates the activity of the *VdPKS1* gene cluster. Importantly, VdPKS9 also regulates the switch between vegetative and dormant states, that is, the natural albinistic hyaline hyphal-type (AH-type) and melanized hyphal-type (MH-type). In summary, these findings reveal a pivotal branch in which VdPKS9 contributes to the regulation of melanin biosynthesis, MS formation, morphology and virulence, and further illustrates the conserved significance of PKS9 in filamentous fungi.

## Results

### *Verticillium dahliae* polyketide synthase 9 (VdPKS9) plays a critical role in pathogenicity

**Fig. 1:**
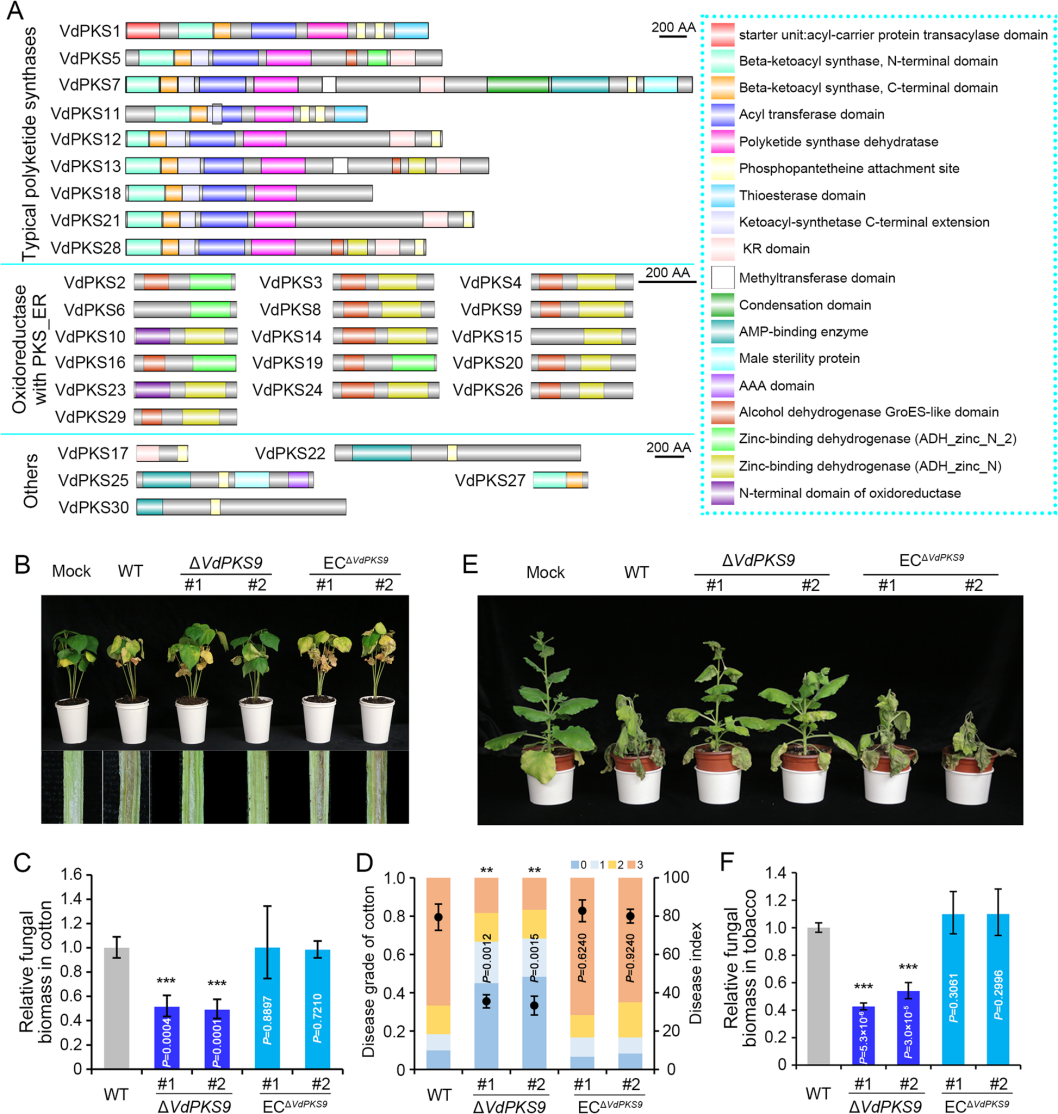
*Verticillium dahliae VdPKS9* encodes a quinone oxidoreductase homolog critical for pathogenicity. **A** Conserved domain structures of 30 predicted VdPKSs in *V. dahliae*. The conserved domains were predicted by multiple pipelines of SMART, InterPro, and Pfam and labeled in different colors. The PKSs were divided into three sub-families by the different characteristics of conserved domains, including typical fungal PKSs, oxidoreductases, and others. AA, amino acids. **B** Pathogenicity assay to investigate the role of the PKSs member VdPKS9 in virulence of *V. dahliae*. Cotton plants were mock-inoculated (Mock) or inoculated with wild-type strain AT13 (WT), two *VdPKS9* deletion strains (Δ*VdPKS9*), and two corresponding ectopic transformants (EC^Δ*VdPKS9*^), and Verticillium wilt symptoms (top) and vascular discoloration were photographed at 21 days post inoculation (dpi). **C** Quantification of the fungal biomass in cotton shoots by quantitative PCR following inoculation of the indicated strains. Shoot samples were collected from the stem base of plants at 21 dpi. The *V. dahliae* elongation factor 1-α (*EF-1α*) was used as the target of DNA amplification to quantify fungal colonization, and the cotton *18S* gene served as an endogenous plant control. Error bars represent standard errors of the mean. ^***^Significant at *P* < 0.001 (one-way ANOVA). **D** The disease rating associated with each of the different strains was classified as Grade 0 (0–25% leaves wilting), Grade 1 (25–50% leaves wilting), Grade 2 (50–75% leaves wilting), and Grade 3 (75–100% leaves wilting). The ratings were conducted with 20 cotton seedlings at 21 dpi with each of the respective *V. dahliae* strains. The result was analyzed with three replicates of cotton pathogenicity tests. **E** Pathogenicity assay of wild-type strain AT13 (WT), two *VdPKS9* deletion strains (Δ*VdPKS9*), and two corresponding ectopic transformants (EC^Δ*VdPKS9*^) on tobacco plants. Verticillium wilt symptoms were photographed at 14 dpi. **F** Quantification the fungal biomass in tobacco shoots by quantitative PCR. Shoot samples were collected from the stem base of plants at 14 dpi. The tobacco *EF* gene served as an endogenous plant control. Error bars represent standard errors. ^***^P < 0.001 (one-way ANOVA). Pathogenicity was analyzed with three replicates of 20 cotton and 6 tobacco plants, and the fungal biomass was calculated by three independent biological replicates. Error bars represent SD (standard deviations) of 3 independent repeated measurements, and asterisks indicate a significant difference

The composition of the PKS family members in *V. dahliae* (hereafter called VdPKSs) was investigated by analyzing the conserved domains in the predicted proteome of strain Vd991 [38]. In total, 30 VdPKSs (VdPKS1–VdPKS30) were identified that contain at least one conserved domain that potentially is involved in SM (Additional file 1: Table S1). The characteristics of VdPKSs were further defined by multiple conserved domain prediction pipelines, including SMART, InterPro, and Pfam database, which showed that VdPKSs can be classified into three types: typical PKSs (nine members), oxidoreductase-containing PKS_ER domain (16 members), and others (five members) (Fig. 1A). To determine the role of the putative PKSs in pathogenicity, the *VdPKSs* were independently deleted in the highly virulent strain AT13. With the exception of *VdPKS2*, *VdPKS20*, and *VdPKS27*, polymerase chain reaction (PCR) detection showed that 27 *VdPKSs* were successfully deleted for this study. Two independent deletion mutant strains of each of the 27 *VdPKSs* deletion mutants were evaluated for pathogenicity (Additional file 2: Figure S1). The mutants of five *VdPKSs* had compromised virulence on cotton (*P* < 0.05) (Additional file 2: Figure S2), including *VdPKS7* (lovastatin nonaketide synthase), *VdPKS9* (quinone oxidoreductase, *P* < 0.01), *VdPKS14* (alcohol dehydrogenase), *VdPKS25* (*L*-aminoadipate-semialdehyde), and *VdPKS30* (dehydrogenase and acyl-CoA synthetases) (Additional file 1: Table S1). Especially, deletion of the *VdPKS9* clearly decreased Verticillium wilt symptoms (foliar wilting and vascular discoloration) and significantly reduced the ability of hyphae to proliferate (fungal biomass) in shoots in infected cotton plants. Additionally, the high-virulent phenotype was recovered by complementation of the *VdPKS9* deletion mutant (Fig. 1C, D). *VdPKS9* also contributed to virulence on tobacco (Fig. 1E, F). Together, these results indicated that *V. dahliae* employs PKSs that participate in pathogenicity, and VdPKS9, encoding a homolog of a putative quinone oxidoreductase, is critically important for pathogenicity.

### VdPKS9 is not involved in quinone and abiotic stress responses

**Fig. 2:**
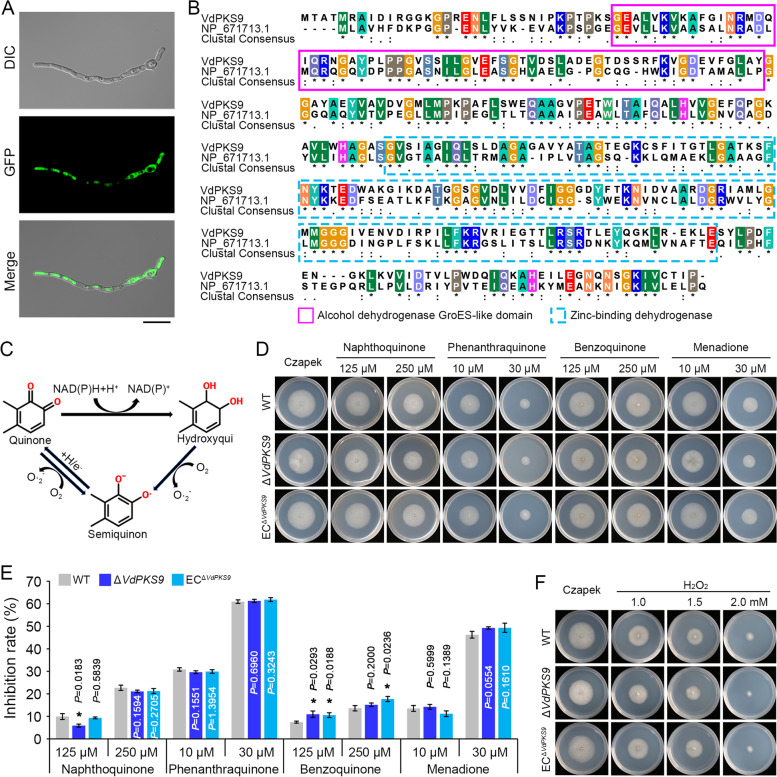
*Verticillium dahliae* VdPKS9 is not involved in quinone and oxidative stress responses. **A** Subcellular localization of VdPKS9 in *V. dahliae*. The VdPKS9-GFP fusion was introduced into the genome of wild-type strain AT13, and the transformant was incubated on hydrophobic coverslips for 24 h. The expression of the VdPKS9-GFP fusion in hyphae and conidia was observed by fluorescence microscope. Scale bar = 20 μm. **B** Sequence alignment of VdPKS9 with human quinone oxidoreductase PIG3 isoform 1. Aligned sequence with the dashed box in purple and blue color represents the alcohol dehydrogenase GroES-like domain and zinc-binding dehydrogenase domain, respectively. Genebank accession of PIG3: NP_671713. **C** Diagram depicting states of quinone transformation in mammals and plants. One-electron transfer of quinone reduction produces a stable free radical anion intermediate: semiquinone. Two-electron reduction of quinone by NAD(P)H-dependent quinone-reducing enzymes yields hydroxyquinone which can also be excreted or oxidized to the semiquinone. In this cycle, the hydroxyquinone and semiquinone states oxidized by molecular oxygen to form superoxide anions that maintain ROS generation [73]. **D**, **F** Colony morphology of *V. dahliae* strains growth on medium supplemented with quinones and oxidative compounds. Wild-type strain AT13 (WT), *VdPKS9* deletion strains (Δ*VdPKS9*), and corresponding ectopic transformants (EC^Δ*VdPKS9*^) were cultured on Czapek medium that supplemented with quinones (125 and 250 μM 1,2-naphthoquinone and 1,4-benzoquinone, as well as 10 and 30 μM 9,10-phenanthrenedione and menadione) and hydrogen peroxide (H_2_O_2_). The phenotypes were photographed 7 days after incubation at 25 °C. **D** Medium supplemented with quinones compounds. **F** Medium supplemented with H_2_O_2_. Error bars represent standard errors of at least 3 independent repeated measurements, and each experiment was replicated three times. ^*^P < 0.05 (one-way ANOVA). **E** Medium supplemented with quinone compounds

*VdPKS9* encodes a protein of 338 aa (amino acids) with a functional homolog of quinone oxidoreductase (Additional file 1: Table S1). Sequence analysis showed that VdPKS9 shares high sequence identities with other fungal proteins from *C. aenigma* (68.47%), *B. cinerea* (59.59%), and *Beauveria bassiana* (63.42%) and retains two conserved domains including an alcohol dehydrogenase GroES-like motif and a zinc-binding dehydrogenase domain (Additional file 2: Figure S3). VdPKS9 was localized in the cytoplasm in both hyphae and conidia when fused with the green fluorescent protein (GFP) (Fig. 2A), consistent with a role as an oxidoreductase. Interestingly, VdPKS9 also contained the conserved domains of NADH quinone oxidoreductase of the human p53-inducible gene 3 (PIG3) (Fig. 2B). PIG3 shows significant homology to NADH quinine oxidoreductase 1 (NQO1) that catalyzes the two-electron reduction of ortho-quinone [73, 74], and is widely recognized as a marker of apoptosis [75, 76], suggesting that VdPKS9 may function similarly to PIG3 to counter the cytotoxicity induced by quinones or oxidative stress (Fig. 2C). Thus, the sensitivity to the quinones or oxidative stressors were tested in the *VdPKS9* deletion mutants. However, the *VdPKS9* deletion mutants displayed similar levels of growth inhibition as the wild-type strain and the ectopic transformants in response to stress from several quinones or oxidative compounds, including ortho-quinones (1,2-naphthoquinone and 9,10-phenanthraquinone), para-quinones (1,4-benzoquinone and menadione), and hydrogen peroxide (H_2_O_2_) (Fig. 2D–G). Additionally, deletion of *VdPKS9* also did not affect the sensitivity of the mutants to osmotic stress (supplemented with sorbitol) and cell wall integrity stress (supplemented with Congo red or sodium dodecyl sulfate) (Additional file 2: Figure S4). Thus, VdPKS9 likely does not function as a quinone oxidoreductase or in response to these abiotic stresses.

### VdPKS9 negatively regulates the melanin biosynthesis pathway

**Fig. 3:**
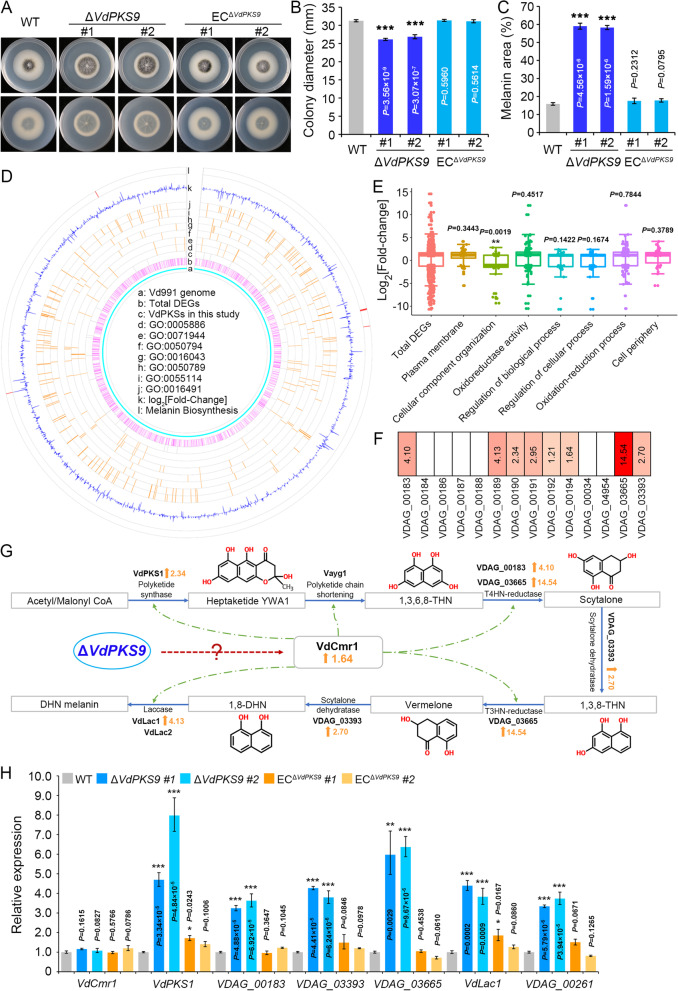
VdPKS9 is necessary for radial growth and negatively regulates melanin biosynthesis in *Verticillium dahliae*. **A** Investigation the colony morphology of indicated strains. Wild-type strain AT13 (WT), two *VdPKS9* deletion strains (Δ*VdPKS9*), and two corresponding ectopic transformants (EC^Δ*VdPKS9*^) were cultured on potato dextrose agar (PDA) medium. Pheotypes were photographed 7 days after incubation at 25 °C in dark. **B**, **C** Measurements of the colony diameter and melanin area of the indicated strains in panel **A**. Error bars represent standard errors of three independent repeated measurements. ^***^P < 0.001 (one-way ANOVA). **B** Colony diameter, **C** melanin area. Each experiment was replicated three times. **D** Transcriptome comparison of *VdPKS9* deletion mutant compared to the wild-type strain. The plot indicates the DEGs, GO enrichment with significance (*P* < 0.005, Pearson chi-square test), melanin biosynthesis-related genes, and genes encoding PKSs in *V. dahliae*. Circle k represent the fold-change of DEGs, bars distribute to the top and bottom side represent up- and downregulation, respectively. **E** Analysis the significance GO items of *VdPKS9* deletion mutant compares to wild-type strain. The significance was calculated by the values of fold-change in each GO items compare to total DEGs. ^**^P < 0.01 (one-way ANOVA). **F** The levels of upregulated melanin pathway genes in Δ*VdPKS9* strain. **G** Diagram of the melanin biosynthesis pathway in *V. dahliae* and the putative point at which VdPKS9 may negatively regulate the pathway. The melanin biosynthesis pathway was drawn from a previous study [12]. **H** Analyses of the relative expression of melanin biosynthesis genes in *VdPKS9* deletion mutant were compared to those of the wild-type strain and ectopic transformants by reverse transcription-quantitative PCR (RT-qPCR). Samples were collected 5 days after incubation in Czapek medium at 25 °C in the dark. The expression levels of each gene were calculated by RT-qPCR using the 2^−ΔΔCT^ method. The expression levels were calculated in three replicates. Error bars represent standard errors of the mean, ^*^P < 0.05, ^**^P < 0.01, and ^***^P < 0.001 (one-way ANOVA)

The potential role of VdPKS9 in growth and morphology of *V. dahliae* was investigated on nutritionally rich potato dextrose agar (PDA). Radial growth was inhibited by deletion of *VdPKS9* and was recovered by complementation of the *VdPKS9* deletion mutants (Fig. 3A). The *VdPKS9* deletion mutant was significantly reduced in its vegetative growth compared to the wild-type strain (Fig. 3B). Remarkably, melanin accumulation was significantly enhanced in *VdPKS9* deletion mutants compared to that produced by the wild-type strain and complemented transformants (Fig. 3A, C). These results strongly suggested *VdPKS9* is involved in vegetative growth and in the negative regulation of melanin accumulation. Next, the transcriptome of the *VdPKS9* deletion mutant was compared to that of the wild-type strain to investigate how the *VdPKS9* affects the vegetative growth and melanin accumulation. In total, deletion of *VdPKS9* resulted in 1330 differentially expressed genes (fold-change ≥ 2.0, *P* < 0.01, 843 DEGs upregulated) under the normal growth on Czapek (Czapek-Dox) medium (Additional file 1: Table S4). Gene ontology (GO) analysis revealed that the DEGs were enriched in six GO terms with significance (*P* < 0.005, Pearson chi-square test), including plasma membrane (GO:0005886), cell periphery (GO:0071944), cellular component organization (GO:0016043), and oxidation-reduction process (GO:0055114) (Fig. 3D). The types of terms associated with those upregulated genes may affect morphological development and other biological processes. The genes associated with the cellular component organization were present in a higher downregulation pattern in the *VdPKS9* deletion mutant (Fig. 3E; Additional file 1: Table S4). DEGs enriched in the oxidation-reduction process may be anticipated considering the function of homologs of similar PKSs involved in oxidation-reduction processes and secondary metabolite biosynthesis (Fig. 3D). Other members of the gene cluster involved in melanin biosynthesis were significantly upregulated in the *VdPKS9* deletion mutant (Fig. 3F). Gene expression assays confirmed that many of the genes involved in melanin biosynthesis with the exception of the transcription factor VdCmr1, were significantly upregulated in *VdPKS9* deletion mutants compared to the wild-type strain and complemented transformants (Fig. 3G). These results were consistent with the high melanin accumulation in the *VdPKS9* deletion mutants (Fig. 3A). We thus concluded that VdPKS9 negatively regulates the melanin biosynthesis pathway to affect melanin accumulation during vegetative growth.

### VdPKS9 negatively regulates the development of MS

**Fig. 4:**
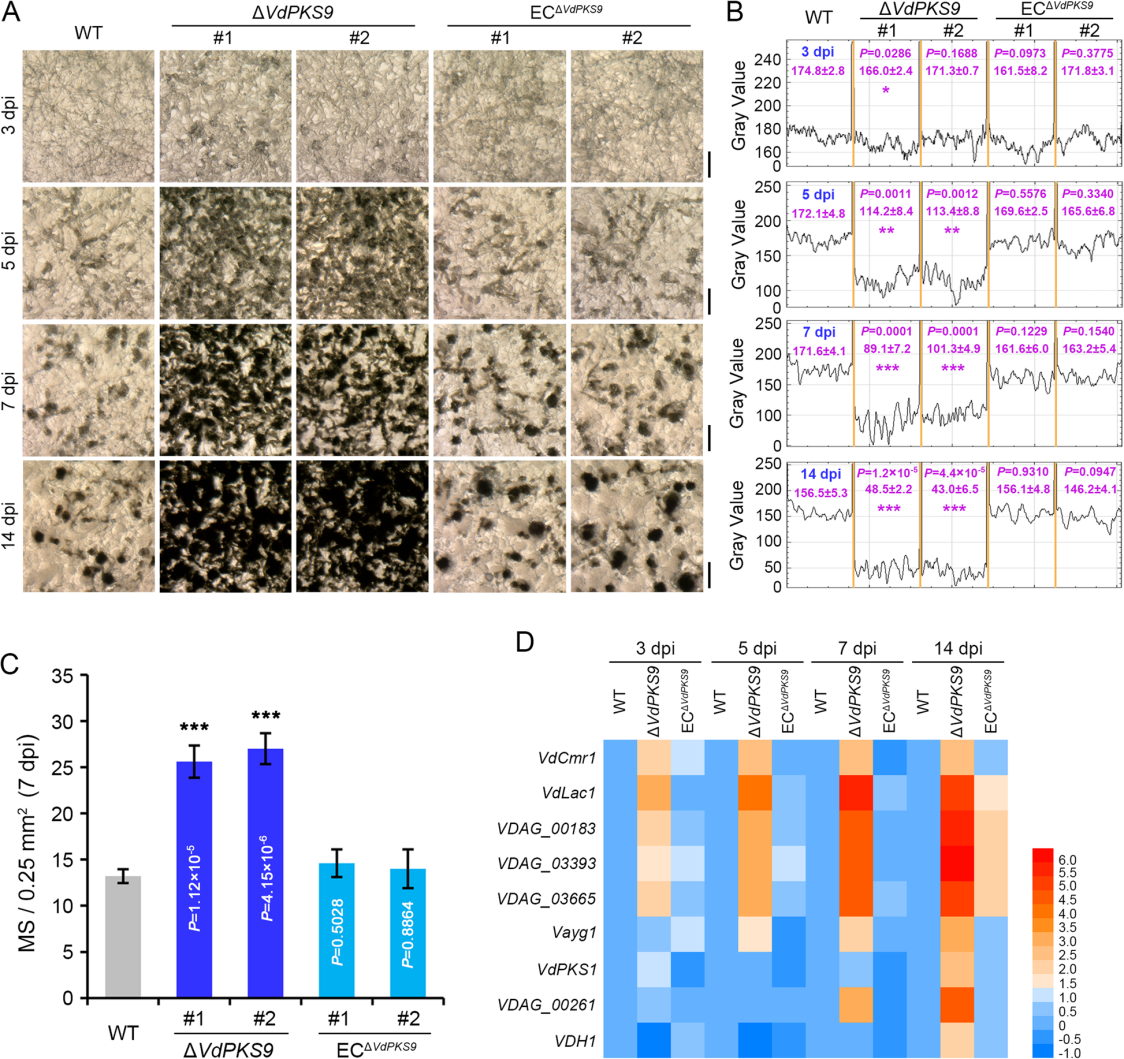
*Verticillium dahliae* VdPKS9 is a negative regulator of both MS development and melanin biosynthesis. **A** Analyses of MS formation. The conidia suspensions (1.0 × 10^6^ conidia/mL, 60 μL) of wild-type strain AT13 (WT), two *VdPKS9* deletion strains (Δ*VdPKS9*), and two corresponding ectopic transformants (EC^Δ*VdPKS9*^) were spread on the BMM medium plates that pre-covered with cellophane membranes. The development of MS was observed at 3, 5, 7, and 14 days after incubation at 25 °C in the dark and photographed with a stereoscope. The experiment was replicated three times independently for each strain, and at least three plates were observed each time. Scale bar =100 μm. **B** Quantification of melanin deposition in visual field by color density. Melanin accumulation level was evaluated by the density of gray color using ImageJ scanning. Three random visual fields were selected for color density scanning, ^*^P < 0.05, ^**^P < 0.01, and ^***^P < 0.001 (one-way ANOVA). **C** Statistics on the numbers of mature MS. *VdPKS9* deletion strains (Δ*VdPKS9*), corresponding ectopic transformants (EC^Δ*VdPKS9*^), and the wild-type strain AT13 (WT) was incubated in BMM medium, and the number of MS was calculated 7 days after incubation on 25 °C in dark. Six independent visual fields of 0.5 mm squared were used in calculations. Error bars represent the standard deviation, and each experiment performed three repeats, ^***^P < 0.001 (one-way ANOVA). **D** Analyses of the relative expression of melanin biosynthesis genes during the MS development among *VdPKS9* deletion strains (Δ*VdPKS9*), corresponding ectopic transformants (EC^Δ*VdPKS9*^), and the wild-type strain AT13 (WT). Samples were collected indicated time-point after incubation in BMM medium at 25 °C in the dark. The expression levels of each gene were calculated by RT-qPCR for 3 repetitions using the 2^−ΔΔCT^ method. The heatmap of melanin biosynthesis gene expression patterns were normalized by the value Log_2_ (fold-change)

A previous study suggested that the VdSho1 signaling pathway that contributes to melanin biosynthesis also contributes to host cell penetration [62]. Thus, as the *VdPKS9* mutant exhibited increased melanin accumulation, the *VdPKS9* mutant was examined for its role in penetration of cellophane membranes, simulating penetration of the host epidermis. The results showed that the *VdPKS9* deletion mutant displayed similar ability to penetrate cellophane membrane as the wild-type strain and ectopic transformants at four different incubation times, and displayed the penetration phenotype at 64 h after incubation on the membrane (Additional file 2: Figure S5). This suggested that the pathogenicity function of VdPKS9 is independent of penetration and confirms previous findings that melanin itself is not required for pathogenicity [12]. The role of VdPKS9 in MS formation was systematically investigated as melanin biosynthesis is recognized as tightly coupled with this process. The *VdPKS9* deletion mutant displayed high density MS at 7 days after incubation on the MS inducing buffered minimal methanol (BMM) medium, but only a few MS were present in the wild type and the complemented transformants (Additional file 2: Figure S6). Further, the progress of MS formation from early to later stages was investigated at four incubation times on BMM medium. Almost all hyphae of the *VdPKS9* deletion mutants were swollen and the microsclerotial initial structures covered the entire surface 3 days after incubation, compared to the relatively smooth hyphae of wild-type and ectopic transformants (Fig. 4A). Subsequently, *VdPKS9* deletion mutants began to agglutinate the precursors of moniliform MS with uniform melanization but accumulated only limited foci of pigmentation in the wild-type strain at 5 days after incubation on BMM medium (Fig. 4A). Although mature MS formed in all strains at 7 dpi, the MS density was significantly higher in the *VdPKS9* deletion mutants compared with the wild-type strain and complemented transformants (Fig. 4A, B). The *VdPKS9* deletion mutants nearly lacked vegetative hyphae at 14 days after incubation on BMM medium (Fig. 4A, B). Statistical analysis of the MS number showed that *VdPKS9* deletion mutants had significantly higher (~ 2-fold) MS compared with the wild-type strain and the complemented transformants (Fig. 4C). Predictably, the promotion of MS formation in the *VdPKS9* mutant was also linked with the gene expression level of melanin biosynthesis-related genes, which gradually increased with incubation time (Fig. 4D). Together, these results suggested that VdPKS9 acts as a strong negative regulator of the development of MS and melanin biosynthesis in *V. dahliae*.

### VdPKS9 functions as a negative regulator epistatic to the known melanin biosynthesis pathway in *Verticillium dahliae*

**Fig. 5:**
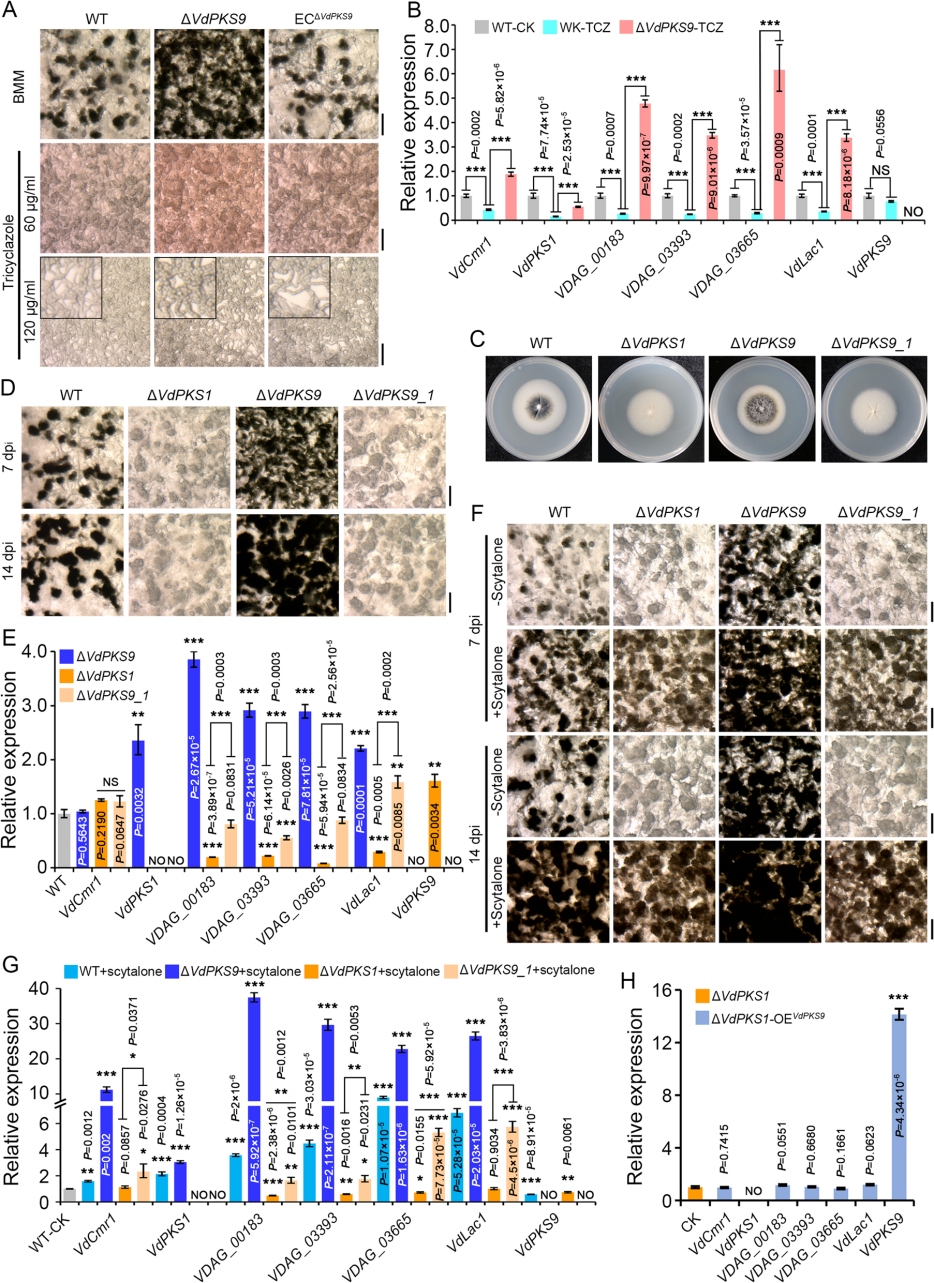
VdPKS9 functions epistatic to the characterized melanin biosynthesis pathway to negatively regulate melanin biosynthesis and MS formation. **A** Investigation of MS formation in *VdPKS9* deletion strains (Δ*VdPKS9*), corresponding ectopic transformants (EC^Δ*VdPKS9*^), and the wild-type strain AT13 (WT) inhibits the melanin biosynthesis pathway using tricyclazole. The MS induction on BMM medium contained indicated concentration of tricyclazole for 7 days and were observed using a stereoscope. **B** Relative expression analyses of the melanin biosynthesis genes in the indicated strains following treatment with tricyclazole. The samples in panel **A** in this figure were collected to extract RNA for reverse transcription-quantitative PCR (RT-qPCR) for analyses of the expression of melanin biosynthesis pathway-related genes. The wild-type strain AT13 was grown on BMM medium without supplementation as the control (WT-CK tricyclazole). The expression levels of each gene were calculated by RT-qPCR using the 2^−ΔΔCT^ method. Error bars represent standard errors of the mean, ^***^P < 0.001 (one-way ANOVA). **C** Colony morphology of the *VdPKS9*::*VdPKS1* double deletion mutant. The indicated strains were cultured on PDA medium for 7 days at 25 °C in the dark. **D** Analyses of MS formation in the *VdPKS9*::*VdPKS1* double deletion mutant. All strains (*VdPKS9* deletion strains Δ*VdPKS9*, *VdPKS1* deletion strains Δ*VdPKS1*, additional deletion of *VdPKS9* under the *VdPKS1* deletion mutant Δ*VdPKS9_1*, and the wild-type strain AT13) were cultured on BMM medium for 7 and 14 dpi, and the MS were photographed using a stereoscope. **E** Analyses of the expression of the melanin biosynthesis genes in the double deletion mutant Δ*VdPKS1::VdPKS9*. The indicated strains were cultured on BMM medium for 7 days. Error bars represent standard errors of the mean, ^**^P < 0.01, and ^***^P < 0.001 (one-way ANOVA). **F** MS morphology of the indicated strains supplemented with the melanin biosynthesis pathway intermediate scytalone. All of the indicated strains were cultured on the BMM medium or supplemented with the scytalone (50 mg/mL) for 7 and 14 days. The MS were observed using a stereoscope, scale bar = 100 μm. **G** Relative expression analyses of the melanin biosynthesis genes of the indicated strains following supplementation with scytalone. The indicated strains were cultured on BMM medium for 5 days. The expression levels of each gene were calculated by RT-qPCR using the 2^−ΔΔCT^ method. Error bars represent standard errors of the mean, ^*^P < 0.01, ^**^P < 0.01, and ^***^P < 0.001 (one-way ANOVA). **H** Relative expression analyses of melanin biosynthesis genes and *VdPKS9* in the Δ*VdPKS1*-OE^VdPKS9^ strain compared with the Δ*VdPKS1* strain. Samples were collected from Czapek medium 5 days after incubating at 25 °C in the dark. The observation of MS and colony melanin, as well as quantitative detection of genes, were repeated three times independently, and three plates or samples were performed each time

Though VdPKS9 is a PKS with homology to a functional quinone oxidoreductase that negatively regulates melanin biosynthesis, the pathway or interacting partners that regulate this response required further investigation. The fungicide tricyclazole can affect melanin production and MS formation by inhibiting the activity of reductases that mediate melanin biosynthesis in fungi [62, 77]. Thus, the interference of melanin biosynthesis and MS formation by tricyclazole treatment was determined in *VdPKS9* deletion mutant, to check if VdPKS9 is an inherent member in the melanin biosynthesis pathway. Although tricyclazole inhibited melanin accumulation in the *VdPKS9* deletion mutant in comparison with the wild type and complemented transformants, the *VdPKS9* deletion mutant displayed higher pigment deposition on the BMM plates supplemented with low concentration tricyclazole (60 μg/mL), and more compact but swollen dormant precursors under the high concentration of tricyclazole (120 μg/mL) (Fig. 5A). Melanin biosynthesis genes were significantly reduced in their expression by the tricyclazole treatment but did not affect the negative regulation of melanin biosynthesis-related genes in the *VdPKS9* deletion mutant (Fig. 5B). Thus, VdPKS9 may exert its regulatory effect epistatic to the *VdPKS1*-associated melanin biosynthesis pathway. Since deletion of *VdPKS1* (key and initial gene of melanin biosynthesis) results in blocking melanin accumulation and albino MS formation [12], we further investigated the relationship between *VdPKS1* and *VdPKS9. VdPKS1* was deleted in the *VdPKS9* mutant background to determine the genetic relationship of VdPKS9 with the known *VdPKS1*-associated melanin biosynthesis pathway. Interestingly, deletion of *VdPKS1* in the *VdPKS9* mutant displayed a phenotype similar to the *VdPKS1* deletion mutant with defects in melanin accumulation and production of albino MS (Fig. 5C, D), but the negative regulation of melanin biosynthesis genes was preserved in the absence of *VdPKS9* in *V. dahliae* (Fig. 5E). Deletion of either *VdPKS9* or *VdPKS1* resulted in the upregulation of gene expression levels of one another (Fig. 5E). These results again suggested that *VdPKS9* is not an inherent member of the known melanin biosynthesis pathway [12]. Subsequently, melanin biosynthesis and MS formation were investigated in cultures supplemented with the melanin intermediate scytalone. As expected, the *VdPKS1* mutant recovered the ability to accumulate melanin and form mature MS on medium supplemented with scytalone (50 μg/mL). Melanin accumulation and MS formation in *VdPKS9* deletion mutant were further enhanced by scytalone supplementation (Fig. 5F; Additional file 2: Figure S7). Correspondingly, the negative regulation by VdPKS9 of melanin biosynthesis genes and higher melanin accumulation was retained in the *VdPKS1* and *VdPKS*9 double deletion mutant (Fig. 5G). Finally, overexpression of *VdPKS9* in the *VdPKS1* deletion background exhibited a phenotype similar to the *VdPKS1* mutant (melanization-defective colonies) (Fig. 5H; Additional file 2: Figure S8), which suggested that *VdPKS9* is not a member of the same melanin biosynthesis pathway mediated by *VdPKS1*. Taken together, VdPKS9 negatively regulates melanin biosynthesis, even in the presence of scytalone or in response to tricyclazole. Thus, VdPKS9 acts epistatic to the known melanin biosynthesis pathway as a negative regulator of melanin biosynthesis, and also strongly negatively regulates MS formation.

### VdPKS9 functions as a regulator of vegetative growth and MS formation in *Verticillium dahliae*

**Fig. 6:**
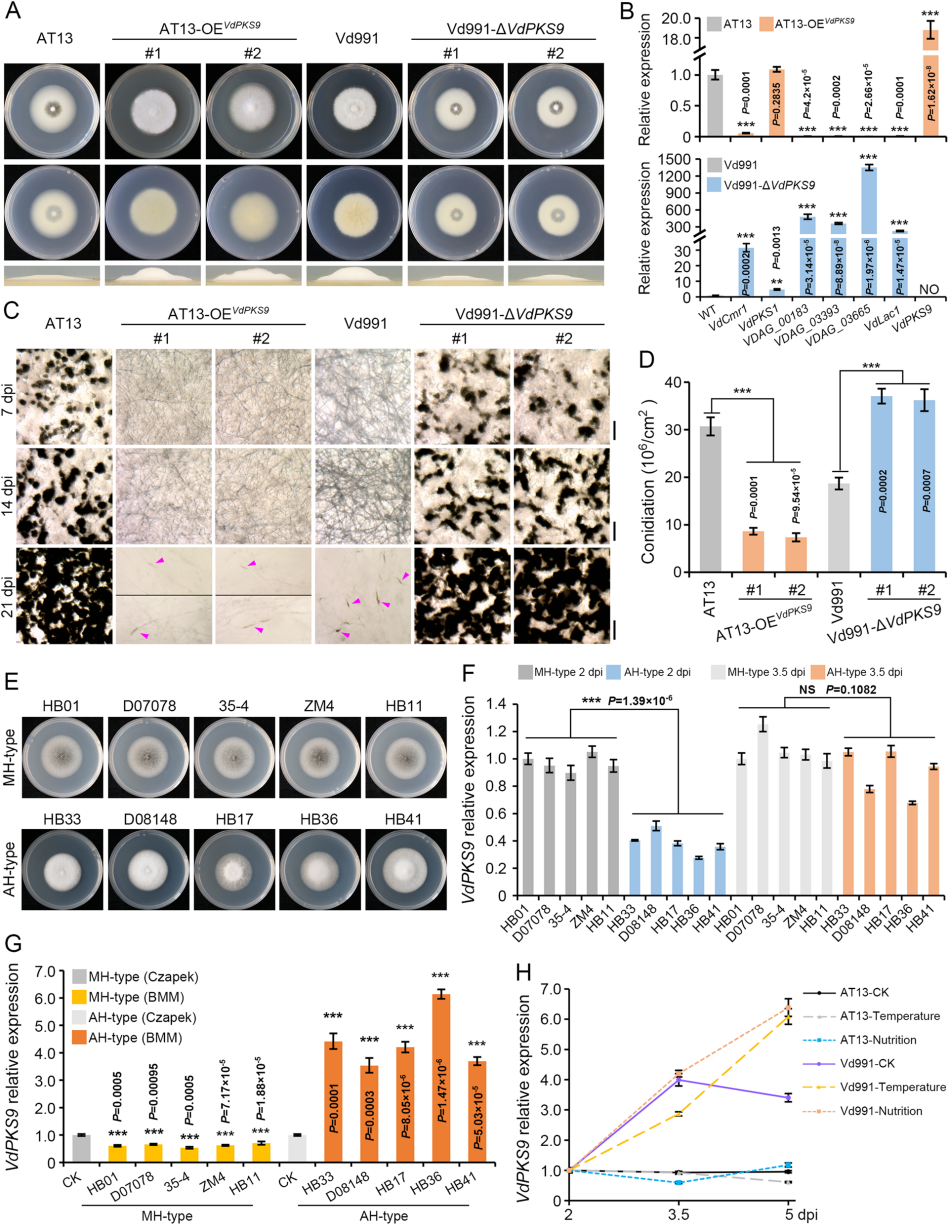
VdPKS9 regulates hyphal growth and MS formation in *Verticillium dahliae*. **A** VdPKS9 mediates the morphological development of hyaline-hyphae in *V. dahliae*. Growth phenotype of wild-type strain (AT13), transformant overexpressing *VdPKS9* in strain AT13, wild-type strain (Vd991, hyaline-hyphae type, highly virulent on cotton), and its *VdPKS9* deletion mutant were cultured on PDA medium. The growth phenotype and the thickness of aerial hyphae were photographed at 7 and 5 days after incubation at 25 °C in dark, respectively. **B** Analyses of the relative expression of melanin biosynthesis genes in the transformant overexpressing *VdPKS9* in strain AT13 and the *VdPKS9* deletion mutant in Vd991 strain by RT-qPCR. RNA samples were collected from the indicated strains that grow on PDA medium for 5 days, and the transcript level in two wild-type strains (AT13 and Vd991) was set as control. **C** Analyses of MS development of two wild-type strains of AT13 and Vd991, AT13 overexpressing *VdPKS9*, and the *VdPKS9* deletion mutant in the Vd991 strain background. All strains were cultured on BMM medium for 7, 14, and 21 days on BMM medium, and photographed by stereomicroscope. Scale bar = 100 μm. Arrowheads indicate MS. Morphological observation showed that each strain had 3 plates and repeated 3 times independently. **D** Analyses of conidiation in the indicated strains. Three 0.5 mm diameter mycelial plugs were collected with a hole puncher from the edge of strains grown for 7 days, and then shaken in sterile water containing 0.1% Tween-20 for 1 min. Conidia were counted using a hemocytometer, with three repeats for each strain. **E** The hyaline hyphae (AH) and melanized hyphae (MH) types in *V. dahliae* strains. Each strain was grown on minimal media plates in the darkness at 25 °C for 7 days. **F** The relative expression of *VdPKS9* between AH-type and MH-type strains during culture on Czapek medium. The conidia of these strains were grown at 25 °C and samples were collected at 2 and 3.5 days, the expression of *VdPKS9* in MH-type strain (HB01) was set as control. Analyses of expression levels in three different experiments were conducted by RT-qPCR using the 2^−ΔΔCT^ method. **G** Relative expression of *VdPKS9* in AH-type and MH-type strains under nutrition stress. The conidia of indicated strains were grown on Czapek medium at 25 °C for 2 days, then transferred to basal medium and incubated an additional 1.5 days. Samples were collected after culturing each media, and the expression levels of *VdPKS9* at 2 days after incubation on Czapek medium for each strain was set as control. Analyses of expression levels in three different experiments were conducted by RT-qPCR using the 2^−ΔΔCT^ method. **H** The expression of *VdPKS9* in AT13 and Vd991 strains in response to stress conditions. The conidia of AT13 and Vd991 strains were cultured on Czapek medium at 25 °C for 2 days, then transferred to basal medium or low temperature (4 °C) and incubated another 1.5 days (2–3.5 dpi), and re-transferred toCzapek medium for an additional 1.5 days. Samples at different growth time points were collected for transcript analysis, and the expression of *VdPKS9* on Czapek medium at 2 days was set as control. Analyses of expression levels in three different experiments were conducted by RT-qPCR with three repetitions using the 2^−ΔΔCT^ method

The above evidence suggested that VdPKS9 negatively affects MS formation, and thus we hypothesized that *VdPKS9* plays a positive role in vegetative growth. Indeed, overexpression of the *VdPKS9* in the wild-type melanization-hyphae strain AT13 strongly enhanced hyphal development without melanin accumulation (Fig. 6A). Deletion of the *VdPKS9* in the hyaline hyphae AH-type strain (Vd991) converted this strain to the MH-type that reduced hyphal development (Fig. 6A). Coincidently, overexpression of the *VdPKS9* significantly suppressed the melanin biosynthesis genes examined, and deletion of *VdPKS9* in the strain Vd991 background significantly upregulated the same set of melanin biosynthesis genes (Fig. 6B). Interestingly, the function of *VdPKS9* in negatively regulating melanin biosynthesis is conserved in fungi that have the *VdPKS9* homolog. *B. cinerea*, *C. gloeosporioides* and *C. fructicola* produce pigment (DHN melanin) (Additional file 2: Figure S9A and S9B), and the *VdPKS9* homolog in each was also activated under the nutrition stress (Additional file 2: Figure S9C). Therefore, the *VdPKS9* homologs in these fungi retained the conserved function in negatively regulated melanin biosynthesis since their overexpression in *V. dahliae* (AT13 strain) reduced melanin deposition by suppressing expression of melanin biosynthesis genes (Additional file 2: Figure S9D and S9E). In addition, overexpression of the *VdPKS9* in the MH-type reduces the ability to induce mature MS and deletion of *VdPKS9* in AH-type quickly promotes the formation of MS (Fig. 6C). Overexpression of *VdPKS9* can significantly compromise the development of conidiation while enhancing hyphal biomass (Fig. 6D and Additional file 2: Figure S10B). Thus, VdPKS9 promotes vegetative growth, and this is correlated with negative regulation of the melanin biosynthesis pathway and MS formation in *V. dahliae*.

Next, we examined whether VdPKS9 affects the morphological development of MH-type or AH-type hyphae in *V. dahliae*. In the *V. dahliae* strains examined, the MH-type and AH-type hyphae are apparent on MM medium after 5 days of growth (Fig. 6E). Gene expression analysis showed that *VdPKS9* enhanced the AH-type more than MH-type from 2 to 3.5 days, although the expression level of *VdPKS9* was higher in the MH-type than the AH-type following incubation for 2 days (Fig. 6F; Additional file 2: Figure S10A). These results also indicated that VdPKS9 positively promotes vegetative growth (AH-type) in stress responses, as *V. dahliae* displayed increased expression of *VdPKS9* when the nutrient consumption was gradually exhausted in later growth stages in culture (Fig. 6F). To confirm that VdPKS9 regulates vegetative growth, the MH-type and AH-type strains were transferred onto BMM (insufficient nutrient medium) after incubating on Czapek medium (sufficient nutrition). Indeed, the expression level of *VdPKS9* was significantly downregulated in MH-type but strongly upregulated in AH-type hyphae after being challenged by nutrition stress (Fig. 6G). Finally, with the exception of nutrition stress, *VdPKS9* expression was consistently upregulated in the hyphae of the AH-type (Vd991) strain, but it was downregulated or not effected in the MH-type (AT13) strain under temperature, ultraviolet light, oxidation, and pH extremes stresses that often induce MS formation (Fig. 6H and Additional file 2: Figure S10C). These results suggested that VdPKS9 functions as a switch that stimulates vegetative growth and suppresses MS formation and this may be influenced by melanin biosynthesis.

### VdPKS9 is epistatic to VdPKS1 and contributes to pathogenicity of *Verticillium dahliae*

**Fig. 7:**
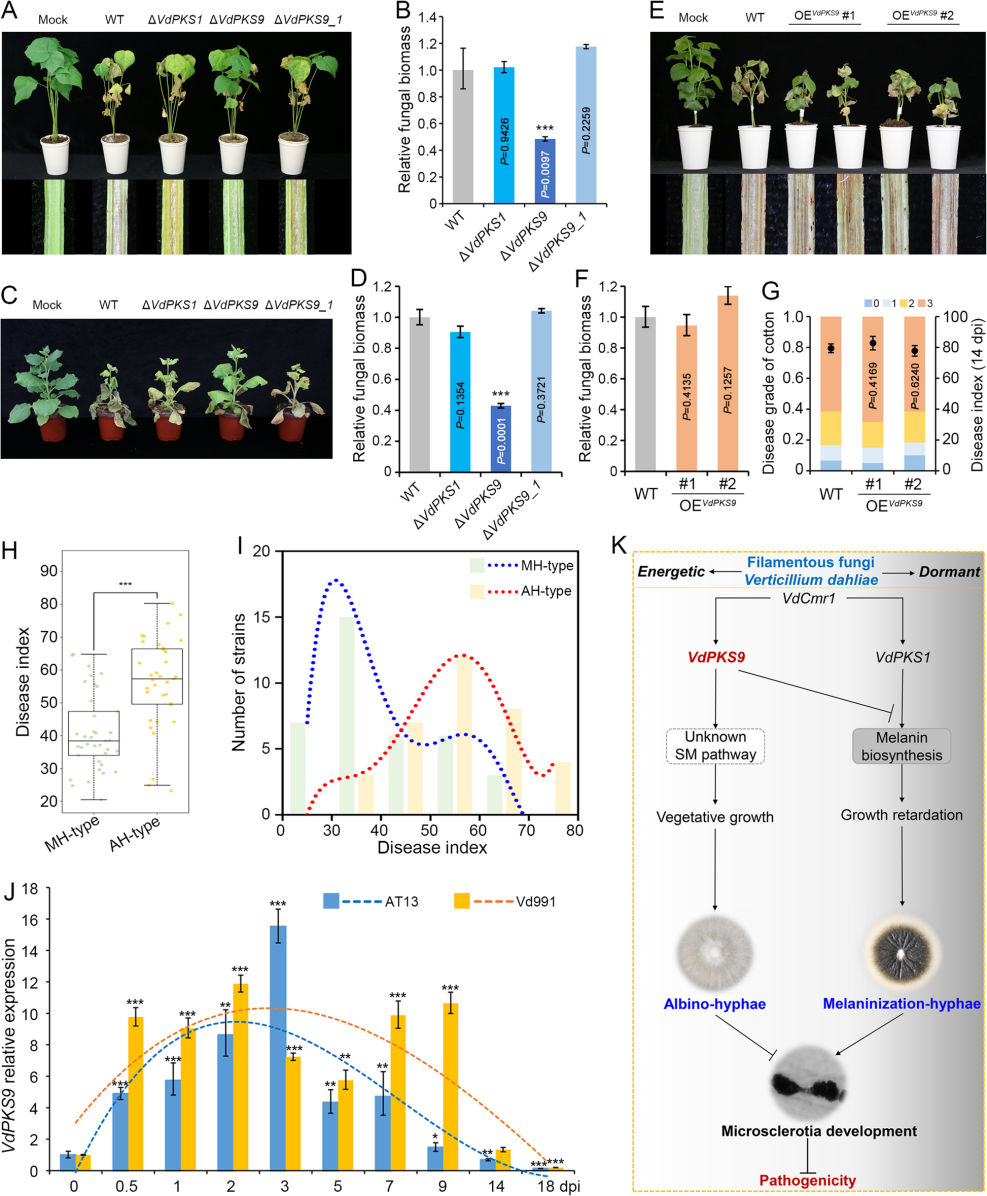
VdPKS9 colludes with VdPKS1 to contribute to pathogenicity of *Verticillium dahliae*. **A**, **C** Pathogenicity assay of the indicated strains on cotton and tobacco. Cotton (**A**) and tobacco (**C**) seedlings were mock inoculated (Mock) or inoculated with wild-type strain AT13 (WT), *VdPKS9* deletion strains (Δ*VdPKS9*), *VdPKS1* deletion strains (Δ*VdPKS1*), and double deletion of *VdPKS9* and *VdPKS1* strains (Δ*VdPKS9_1*), and the Verticillium wilt symptom (top) were photographed at 21 and 14 dpi on cotton (with addition phenotype of vascular discoloration) and tobacco, respectively. **B**, **D** Quantification the fungal biomass in plant shoots by quantitative PCR following inoculation of the indicated strains. Cotton (**B**) and tobacco (**D**) shoot samples were collected from the stem base of plants at 21 dpi. The *V. dahliae* elongation factor 1-α (*EF-1α*) was used to quantify fungal colonization, and the cotton *18S* gene and tobacco *NbEF-1α* served as an endogenous plant control. The experiment was replicated three times. The pathogenicity was analyzed with three replicates of 20 cotton and 6 tobacco plants, and the fungal biomass was calculated by three independent biological replicates. Error bars represent standard errors. ^***^P < 0.001 (one-way ANOVA). **E–G** Pathogenicity assay of transformants overexpressing *VdPKS9* in the wild-type strain AT13. Cotton seedlings were mock-inoculated (Mock) or inoculated with wild-type strain AT13 (WT) and *VdPKS9* overexpressing transformant and evaluated by pathogenicity indexes at 21 dpi, included foliar Verticillium wilt symptoms (top) and vascular discoloration. **E** Fungal biomass development (**F**) and the disease index (**G**). The disease index was analyzed with three replicates of cotton pathogenicity tests. **H** Pathogenicity assay of the AH-type and MH-type strains during infection of cotton plants. In total, 37 AH-type strains and 34 MH-type strains were selected for virulence analysis by root-dip method on cotton plant, and disease indices were evaluated at 28 dpi. **I** Analyses of the pathogenicity characteristics of AH-type and MH-type strains. Polynomial fitting was employed. **J** The relative expression of *VdPKS9* in AH-type and MH-type strains infecting and colonizing cotton plants. RNA of samples collected from 0 to 18 dpi was extracted and reverse transcribed. The expression levels were calculated by RT-qPCR in three replicate experiments. Error bars represent standard errors, ^*^P < 0.01, ^**^P < 0.01, and ^***^P < 0.001 (one-way ANOVA). **K** A working model showing VdPKS9 colludes with VdPKS1 to contribute to pathogenicity of *Verticillium dahliae*. The *V. dahliae* PKS member VdPKS9 functions in an unknown SM pathway that participates in the maintenance of hyphal growth, and thus promotes the pathogenicity. Another PKS, VdPKS1, functions in melanin biosynthesis and accelerates hyphae melanization, a function negatively regulated by VdPKS9. Broadly, *V. dahliae* networks the two PKSs of VdPKS9 and VdPKS1 to contribute to pathogenicity by suppressing MS and melanin production and promoting hyphal growth. This represents the potential mechanism by which the AH-type strains display stronger virulence than MH-type strains in *V. dahliae*

To further determine how *VdPKS9* contributes to pathogenicity by manipulating vegetative growth and MS formation in *V. dahliae*, pathogenicity of the individual deletion mutants of *VdPKS9* or *VdPKS1*, the double deletion of *VdPKS9* and *VdPKS1* deletion mutant (Δ*VdPKS9_1*), and *VdPKS9* overexpressing strain (in AT13) were examined on cotton and tobacco. The results revealed that *VdPKS9* (that suppresses MS) but not *VdPKS1* (MS formation unaffected) contributes to pathogenicity on cotton (Fig. 7A, B). Similar results were observed on tobacco (Fig. 7C, D). The Δ*VdPKS9_1* displayed similar MS formation but was devoid of the dark melanization (Fig. 5D) and was also not impaired in pathogenicity on cotton and tobacco (Fig. 7A–D). Although *V. dahliae* was converted into the AH-type after overexpressing *VdPKS9* in AT13 strain, the transformants did not enhance pathogenicity on cotton (Fig. 7E–G), likely due to the significant reduction in conidiation compared with the wild-type (Fig. 6C, D). These results suggested that pathogenicity of *V. dahliae* is interconnected with MS formation or vegetative growth regulated by *VdPKS9*. To further examine this mechanism, the potential differential pathogenicity of AH-type and MH-type strains were tested on cotton. The AH-type (34 strains) displayed significantly higher virulence than the MH-type (37 strains) (Fig. 7H; Additional file 1: Table S5); most of the MH-type strains had a low disease index (DI) value while most of the AH-type strains had a higher DI value (Fig. 7I). Gene expression analysis clearly showed that the transcript levels of *VdPKS9* were continuously increased in AH-type strain (Vd991) compared with those of the MH-type strain (AT13) during infection of cotton (Fig. 7I). Potentially, this retains vegetative growth under the stresses incurred during infection and colonization, and thus functions in the pathogenicity. Taken together, VdPKS9 is epistatic to VdPKS1 in regulating the development of melanized MS, but VdPKS9 participates in an independent pathway that contributes to virulence by promoting vegetative growth while inhibiting MS development and melanization. This may explain why the AH-type strains displayed higher virulence than MH-type strains in *V. dahliae*.

## Discussion

MS of *V. dahliae* are produced in the later stages of infection or in senescent plant tissues as a response to depleting nutrition [12, 44], and thus play a critical role in the disease cycle of *V. dahliae* [35, 45]. Their formation is accompanied by melanin deposition in *V. dahliae* [42, 58], but melanin production is not always coupled with MS formation and greater pathogenicity [12, 62]. Yet linkages between pathogenicity and MS formation and melanin production have been made in the literatures, but the basis for this linkage is unresolved [44, 55, 62]. By systematically exploring the role of the *V. dahliae* PKSs, we identified VdPKS9, a quinone oxidoreductase homolog in *V. dahliae*, as critically important for pathogenicity. VdPKS9 negatively regulates MS development and melanin biosynthesis while promoting hyphal growth. The deletion of *VdPKS9* results in the upregulated expression of melanin biosynthesis-related genes and lagging growth to increase MS numbers in comparison to the wild type strain, suggesting that VdPKS9 acts as an important regulatory switch that mediates MS production important for its long-term survival, or the hyphal growth necessary for virulence.

Fungal secondary metabolites produced by plant pathogens can act as virulence factors that directly impact crop yields and food safety [23], and the polyketides accompanied by their precursors—PKSs—are important determinants of pathogenicity in many pathogens [23, 78, 79]. Generally, polyketides mediate pathogenicity through several processes such as survival (e.g., melanin), self-defense (e.g., antibiotics), and escape host attack (e.g., phytotoxins) [78]. PKS proteins are traditionally divided into three types. Type I PKSs are multifunctional enzyme systems composed of several modules. Type II PKSs form heterodimers consisting of a KS and a chain length factor and ACPs, while type III PKS only relies on the KS homodimer to catalyze the biosynthesis of products [70]. Based on the differences in domain composition and product catalytic mode, fungal PKSs belong to an iterative type I PKS and have been extensively studied. *Fusarium* possesses a variety of polyketone compounds, and there were 16, 14, 15, and 13 type I PKSs in *F. verticillioides*, *F. oxysporum*, *F. graminearum*, and *F. solani*, respectively [80]. However, *V. dahliae* only contains 9 type I PKSs, and the reactions and products catalyzed by them may be quite different from those of *Fusarium*. Similarly, fewer non-ribosomal peptide synthetases (NRPS) and PKS-NRPS hybrid (3 and 1, respectively) [72] also suggest that *V. dahliae* has unique secondary metabolite spectra. In *V. dahliae*, the secondary metabolites also have a role in pathogenicity [37, 39, 40], but few PKSs have been identified to contribute pathogenicity during infection of host plants and their regulatory mechanisms are unknown. With the large-scale gene deletion of PKS gene family members, we demonstrated that several PKSs contribute to the pathogenicity of *V. dahliae* on cotton (Additional file 2: Figure S1 and S2), and *VdPKS9* being the most critical (Fig. 1).

VdPKS9 possesses the conserved domains of NADH quinone oxidoreductase similar to the human PIG3, which is involved in ROS formation, cancer cell apoptosis, autophagy, DNA repair, and other important biological processes in humans [73, 74, 81]. However, deletion of *VdPKS9* did not affect the multiple stress sensitivities or the self-detoxification of quinones (Fig. 2; Additional file 2: Figure S4), nor did it cause excessive changes in the expression of major known genes responsible for autophagy, programmed cell death, and DNA damage repair in *V. dahliae* (Additional file 2: Figure S11). Thus, how *VdPKS9* is involved in secondary metabolite synthesis is still unknown and requires further study.

*VdPKS9* negatively regulates melanin biosynthesis (Fig. 3) and acts epistatically on the melanin biosynthesis pathway to curtail melanogenesis. DHN-melanin is ubiquitous in many fungi and is produced via a conserved biosynthetic pathway, typically by members of a *PKS* gene cluster, such as in *M. oryzae*, *A. fumigatus*, and *Pestalotiopsis fici* [14, 23, 82]. In *V. dahliae*, genes encoding the DHN-melanin pathway, including *VdPKS1* and the regulatory transcription factor VdCmr1 have been studied [12]. The finding of the additional PKS involved in melanin pathway regulation was unexpected (Fig. 3). Although a branch of melanin pathway results in the synthesis of pentaketide metabolites when inhibited by the tricyclazole, these compounds are extremely unstable and have seldom been isolated from fungi [83]. However, in contrast to the strong inhibition of melanin biosynthesis pathway, *VdPKS9* mutants were not affected by treatment with the DHN-melanin pathway inhibitor, tricyclazole [83, 84] (Fig. 5B), which suggested that *VdPKS9* is not a direct player in the primary pathway for melanin biosynthesis. More importantly, VdPKS9 negatively regulated the melanin biosynthesis-related genes even after interference with melanin biosynthesis via the deletion of *VdPKS1*, supplementation with scytalone, or by inhibition with tricyclazole (Fig. 5). In addition, compared with AT13-OE^*VdPKS9*^, the Δ*VdPKS1*-OE^*VdPKS9*^ strains did not form dense aerial hyphae, nor did they downregulate melanin biosynthesis genes (Fig. 5H; Additional file 2: Figure S8), and virulence was restored in the Δ*VdPKS9_1* strain similar to Δ*VdPKS1* (Fig. 7A–D), and also exhibited a defective pigment deposition in the MS even when exposed to scytalone (Fig. 5F). Although the involvement of biosynthetic pathway and the specific metabolite produced by VdPKS9 is still unknown, the genetic and biochemical evidence clearly demonstrate that VdPKS9 acts epistatic to the known melanin biosynthetic pathway as a negative regulator of melanin biosynthesis in *V. dahliae*.

Melanin is a hydrophobic, high molecular weight pigment with protective properties against stresses, including irradiation, temperature, or reactive oxygen species, and enables fungi to survive adverse environmental conditions [11, 85]. In *V. dahliae*, melanin accumulation is associated with the development of MS that enable *V. dahliae* to survive many years in the soil [12, 41, 58]. Melanin is considered essential for the development of fully functional MS [84]. In this study, we found that the elimination of *VdPKS9* resulted in the acceleration of MS formation, and was synchronous with increased melanin accumulation (Fig. 4; Additional file 2: Figure S6). Conversely, overexpression of the *VdPKS9* resulted the reduced melanin accumulation and less mature MS (Fig. 6C). Undoubtedly, the metabolite produced by VdPKS9 influences the development of MS and, simultaneously, the melanin biosynthesis (Fig. 4). Finally, our study confirms previous findings that MS formation can occur independent of melanin biosynthesis [12, 44, 86] since the MS initials were universally present in the *VdPKS1* deletion mutants (melanin deficient), or in those overexpressing *VdPKS9* (melanin deficient) (Figs. 5D and 6C).

There exist two different types of colonies in *V. dahliae*, namely those of the AH-and MH-types, represented by Vd991 and V592, respectively [87, 88]. In a few cases reported, the AH-type exhibited increased virulence relative to the MH-type [68], but the mechanism is still unknown. Interestingly, VdPKS9 helps retain the AH-type phenotype, presumably by negatively regulating both MS development and melanin biosynthesis (Figs. 3A and 6A). This is especially plausible since deletion of *VdPKS9* significantly impaired vegetative growth (Fig. 3A, B; Additional file 2: Figure S12A), but the growth defect was not caused by the accumulation of toxic intermediates since they rapidly transformed into melanin to promote MS formation (Additional file 2: Figure S12B - S12D). Furthermore, melanized sclerotia of *S. sclerotiorum* and *B. cinerea* are essential for vegetative growth [25, 32], and the level of melanin accumulation is often inversely proportional to the fungal biomass. That is, a decrease in melanin is often associated with increased hyphal growth [89]. A function of PKS9 in the maintenance of vegetative growth was supported by the investigation of a larger population of strains in this study, indicating that the expression of *VdPKS9* was significantly upregulated in AH-type but not in the MH-type under normal growth, and in later developmental stages (Fig. 6E, F). In addition, the AH-type strain strongly expressed *VdPKS9* transcripts when challenged with insufficient nutrition, suboptimal temperature, etc. (Fig. 6G, H; Additional file 2: Figure S10). Indeed, the highly melanized MS of *V. dahliae* germinate and infect plant roots following direct penetration, but more MS are produced in dead plant tissues [90]. Thus, the maintenance of the hyphae is critical following MS germination for infection and colonization. *VdPKS9* exhibited a stronger and more durable expression pattern in AH-type strain than in MH-type strain during infection of cotton (Fig. 7J), which could promote the maintenance of vegetative growth in *V. dahliae*. Broadly, AH-type strains display higher virulence than MH-type strains, possibly due to higher expression levels of *VdPKS9* (Fig. 7H, I) important for the maintenance of hyphal growth. Finally, we demonstrated that the function of VdPKS9 is widely conserved in negatively regulating melanin biosynthesis of filamentous fungi (Additional file 2: Figure S3 and S9). However, overexpression of these homologs did not contribute to the growth of aerial hyphae (Additional file 2: Figure S9). Unexpectedly, overexpression of *VdPKS9* did not result in higher virulence than the wild-type strain, presumably caused by the decline in conidiation in such strains (Fig. 6D). We hypothesize that the downregulation of melanin biosynthesis relieves the constrictions on hyphal growth to facilitate pathogenesis. Similarly, the Sge1 mutants of *V. dahliae* are deficient in conidiation and hyphal growth and show impaired virulence [91]. Since deletion of *VdPKS9* did not affect penetration (Additional file 2: Figure S5), we can conclude that *VdPKS9* contributes to virulence through the retention of hyphal growth by negatively regulating MS and melanin biosynthesis, and may explain why AH-type strains exhibited higher virulence relative to the MH-type strains in *V. dahliae*.

## Conclusions

In conclusion, we characterized VdPKS9 as a novel regulator of melanin biosynthesis and morphological development in *V. dahliae*. VdPKS9 is important for pathogenicity since it suppresses melanin and MS formation while promoting hyphal growth. The role of VdPKS9 homologs is essential in melanin deposition in filamentous fungi. Furthermore, our findings and those of others investigating the survival structures and melanization of *V. dahliae* may help reveal new strategies for the control of devastating diseases caused by these phytopathogenic fungi. The upstream transcription regulators and the putative *VdPKS9* gene cluster, as well as the putative metabolite produced by PKS9 activity, require further investigation.

## Methods

### Fungal strains and growth conditions

The *V. dahliae* strain AT13 was used as the wild type (WT) in this study, which is a defoliating strain isolated from shantung maple in Shandong, China [34]. The wild-type strain was stored in 25% glycerin at −80 °C and was recovered on potato dextrose agar (PDA, 200 g potato, 20 g glucose, and 15 g agar per liter) medium at 25 °C in the dark. Resistant mutants were grown on PDA with hygromycin (50 μg/ml) or geneticin (50 μg/ml).

Colony morphologies and analyses of the growth of hyphae between all strains in response to various stressors were evaluated by incubation on Czapek medium (2 g NaNO_3_, 1 g K_2_HPO_4_, 0.5 g KCl, 0.5 g MgSO_4_, 0.01 g FeSO_4_, 30 g sucrose, 15 g agar and add water to 1 l; sucrose was replaced by 17 g starch, 10 g pectin, or 10 g sodium carboxymethyl cellulose in carbon experiments, respectively) for 7 days. BMM (5 g glucose, 0.2 g NaNO_3_, 0.52 g KCl, 0.52 g MgSO_4_·7H_2_O, 1.52 g KH_2_PO_4_, 3 μM vitamin B1, 0.1 mM vitamin H, 15 g agar, pH 7.5 in 1 l of water) was used for MS formation assays. Protoplasts of *V. dahliae* were mixed with TB3 (200 g sucrose, 10 g yeast extract, 10 g casein acid hydrolysate, 10 g glucose, 8 g agar, and add water to 1 l) medium cooled to 45 °C. The CM, MM, and IM medium were used for collecting conidia suspension, induction of melanin production, and use of the ATMT (*Agrobactirium tumfacience*-mediated transformant) method, respectively, as previously described [92].

### Bioinformatics analysis and characterization of the VdPKSs

Analyses of the genome of in AT13 strain identified PKS-related candidate genes using protein functional domain analysis tools such as InterPro (http://www.ebi.ac.uk/interpro/), Pfam (http://pfam.xfam.org/), and SMART (http://smart.embl-heidelberg.de/), at the threshold of *e*-value <10^−5^. Their functions were described in *V. dahliae* according to the annotated function of the homologs in the National Center for Biological Information (NCBI) database (https://www.ncbi.nlm.nih.gov/) and were named VdPKS1-30 based upon the number in the genome assembly. The structural diagrams of 30 VdPKSs, based upon the domain predictions, were drawn by IBS (Illustrator for Biological Sequences).

The homologs of VdPKS9 were determined by BLASTp of the NCBI Genbank database and were downloaded from Genbank. Multiple sequence alignments were executed using Clustal-W in BioEdit v7.2.0 (http://www.mbio.ncsu.edu/bioedit/), and tree constructed using MEGA 6.0 with NJ method (http://www.megasoftware.net/).

### Generation of gene deletion and complementation mutants

The gene deletion vectors were constructed as follows. First, the 5′- and 3′-flanking regions (0.8–1.5 kb) of the target genes were amplified from genomic DNA of the WT using sequence-specific primer pairs with adapter sequences. The plasmid pDHt2 [93] was linearized by restriction endonuclease *EcoR*I or *Xba*I, and the amplified products were attached to both the ends of the hygromycin resistance gene cassette (*hyg*) by homologous recombination (ClonExpress ® II One Step Cloning Kit, Vazyme, Nanjing, China). The recombinant plasmids were transferred into *Escherichia coli* DH5α competent cells and screened under kanamycin resistance for obtaining positive clones. In addition, the *Xba*I /*EcoR*I-digested pCOM vector [93] with the geneticin (G418) resistance gene was used to construct the complementation vectors, which fused with fragments of genes with native promoter (0.8–1.2 kb) and terminator (0.5–0.8 kb). Subsequently, the deletion and complementation vectors were transformed into *Agrobacterium tumefaciens* strain AGL-1, and the transformation process was performed by ATMT [92], and confirmed for site-specific homologous recombination by diagnostic PCR. Similarly, the double-gene knockout vectors were constructed by linking flanking regions to *Xho*I- and *EcoR*I-linearized pCOM in turn, which were transferred into corresponding single-knockout mutants. The generated transformants were identified by resistance screening and the antibiotic-resistant mutants were subjected to the single-spore purification procedure. Finally, to verify the putative mutants, the diagnostic PCR using internal specific primer pairs of target and resistance genes were carried out. The genomic DNA required in this study was extracted using a DNA isolation mini kit (Vazyme, Nanjing, China), following the manufacturer’s instructions. Primer pairs used are listed in Additional file 1: Table S2.

### Fluorescence microscopy observation for localization of VdPKS9

For generating the GFP fusion cassettes, the RNA of wild-type strain was extracted by RNA kit (Aidlab Biotech, Beijing, China) and reverse transcribed into cDNA using cDNA synthesis Supermix (TransGen Biotech, Beijing, China) following the manufacturer’s instructions, which was used as a template to amplify fragments with 3′- and 5′-UTR regions. The fragments were connected to the commercialized Blunt-zero vector (TransGen Biotech, Beijing, China), transferred into DH5α competent cells, and their insertion screened by antibiotic resistance. The CDS regions without stop codons were amplified and sequenced. The ORF of *VdPKS9* (without stop codon) was cloned into the *Xho*I-linearized plasmid pYF11 encoding *GFP* and geneticin resistance gene by yeast in vivo homologous recombination as previously described [94]. The recombinant plasmids were grown on ampicillin-containing plates for screening and examined by colony PCR to obtain the correct expression vectors. They were subsequently transferred into fungal protoplasts [94] to obtain the GFP-labeled strains.

Protoplast preparation and transformation of *V. dahliae* were modified according to previous reports [94]. Briefly, the AT13 strain was inoculated on PDA plates, incubated for 3 days, cut into mycelium masses, and transferred to 100 ml CM medium in shake culture at low speed. The hyphae were collected, rinsed with sterile distilled water, and digested with enzymatic digestion [94] for 4 h. The released protoplasts were collected, washed, and suspended in 1×STC buffer [94]. The protoplast concentration was adjusted to 5 × 10^6^ /ml. The GFP-fused recombinant plasmids were mixed with 200 μl protoplasts in a solution containing equal volumes of 2 × STC buffer and 1 ml polyethylene glycol (PEG), and the mixture incubated for 10–20 min at room temperature. Next, 3 ml of TB3 culture solution [94] was added to recover the cell wall in low-speed shaking culture about 6 h. The reconstituted protoplasts and the solid TB3 medium were added with G418 after cooling, and used to make plates. All strains were subjected to resistance screening, PCR-based detection, and single spore isolation. Fragment amplification and detection primer pairs are listed in Additional file 1: Table S2. GFP fluorescence was observed using a Leica DM6-B light microscopy system.

### Construction of PKS9 overexpression and heterologous complementary strains

The plasmid pCOM containing the TrpC promotor and G418 resistance was used for obtaining *VdPKS9*-overexpressing and heterologous complementary strains *VdPKS9*, and its homologs were cloned from cDNA of *V. dahliae*, *B. cinerea*, *C. gloeosporioides*, and *C. fructicola*, respectively. The recovered fragments were linked to the *Sac*I/*Xba*I-linearized vector by homologous recombination. Next, the recombinant plasmids were transferred into *E. coli* DH5α competent cells and screened under kanamycin resistance for obtaining positive clones. Plasmids were adjusted to the appropriate concentration for protoplast transformation. To verify the putative mutants, diagnostic PCR and reverse transcription-quantitative PCR (RT-qPCR) for *PKS9* homologous fragments were carried out. Fragment amplification, detection, and detection primer pairs are listed in Additional file 1: Table S2 and S3. The strains and homolog sequences are shown in Additional file 1: Table S5.

### Evaluation of colony morphology, conidiation, membrane penetration, and microsclerotium formation

These experiments referred to previous reports [44, 51, 62]. The WT and mutant strains were cultured on PDA at 25 °C in dark. The edge of each 5-day-old colony was cut into mycelial blocks (1 mm × 1 mm), and inoculated on fresh solid medium. To investigate the quinone oxidoreductase activity of VdPKS9, 1,2-naphthoquinone, 9,10-phenanthrenequione, 1,4-benzoquinone and menadione with different concentrations were mixed into Czapek medium. The H_2_O_2_ of serial concentration gradients as 0.5, 1, 1.5, and 2 mM in Czapek medium were used as the origin of ROS to detect the sensitivity of WT and mutant strains to oxidative stress. In addition, 1 M sorbitol (Solarbio, Beijing, China), 200 μg/ml Congo red (Solarbio, Beijing, China), and 0.01% SDS (Solarbio, Beijing, China) were added in Czapek medium for osmotic stress and cell wall integrity assessment. In addition, vegetative growth and the differences of melanin phenotypes were observed on Czapek salt medium with various carbon sources and PDA plates, respectively. Each media type was inoculated three times independently, and three plates were inoculated per replicate experiment. All these treatments were cultured in the dark at 25 °C, and colony cross diameters were measured at 7 dpi.

The numbers of conidia for propagation were calculated at 7 days after incubation on PDA medium. A hole punch 5 mm in diameter was used to produce mycelial plugs from the periphery of colonies (3 pieces per plate). Conidia were resuspended by vortex oscillation hyphal plugs with sterilized water containing 0.1% Tween 20 (Solarbio, Beijing, China) for 1 min, and the number of conidia was counted using a hemocytometer.

In the simulated penetration experiments, blocks containing hyphae were incubated on MM plates covered with cellophane membranes for 48 h. The membranes were removed every 8 h for a period of 72 hpi. After treatment, the plates were incubated at 25 °C in the dark for another 5 days. To observe the MS formation, the conidia of different strains were harvested from PDA plates and 60 or 10 μl of a conidial suspension (1 × 10^6^/ml) was smeared onto a cellophane membrane (*Ø* = 70 mm, Solarbio, Beijing, China) or 1/6 of a microfiltration membrane (nylon material, *Ø* = 70 mm; pore size = 0.45 μm, Jinlong, Tianjin, China) laid on BMM medium. The development of MS was observed under a stereomicroscope after 3, 5, 7, and 14 days of incubation at 25 °C in the dark. ImageJ was used to calculate the gray value that was simulated from the amount of melanized MS formation, and the smaller the value, the higher the coverage of MS. The above experiments were replicated three times.

### Pathogenicity assay

The 5 × 10^6^/ml spore suspension of each strain was obtained by filtration, harvested by centrifugation, washed, and diluted after shaking in liquid CM medium for 3 days. Susceptible cotton seedlings (Junmian No.1) and *Nicotiana benthamiana* plants grew in the medium of nutrient soil and vermiculite (1:1 v/v) for about 3 weeks, reaching 2 and 9 true leaves respectively. Virulence assays were performed on these seedlings using a root-dip inoculation method [40]. Plants were removed from the soil medium, the soil washed off, and the roots immersed in the conidia suspension for 30 min. Five cotton or 1 tobacco seedling from each group were replanted into a cup. Each transformant was inoculated in 3 replicated experiments with 20 cotton or 6 tobacco seedlings. The WT strain AT13 and water were used as positive and negative controls, respectively. Since cotton begins to show disease symptoms on the 12th–14th day, while tobacco on the 10th day after inoculation, the disease incidence of each group was counted at 21 dpi, and disease severity scores from cotton seedlings were divided into five categories: 0 = healthy; 1 = one true leaf showing yellowing; 2 = two true leaf showing wilt symptoms; 3 = two true leaves fell off and even plant dead (disease index was calculated as Fan et al. [44] described). Subsequently, we removed the plants, washed them, and cut the stem longitudinally to observe the discoloration of the vascular system.

### Analysis of relative gene expression and fungal biomass

All plant/fungal samples were prepared in advance and stored at – 80 °C until use. For example, cotton inoculated with 1 × 10^9^/ml spore suspension of WT was used as the test sample for *VdPKS9* expression in plant infection and colonization, and the roots were collected at 0, 0.5, 1, 2, 3, 5, 7, 9, 14, and 18 dpi. The effects of *VdPKS9* deletion, the expression of melanin biosynthesis genes (*T4HR*, *SCD*, *T3HR*, *VdPKS1*, *VdCmr1*, *VdLac1*, and *VDAG_00261* shown in Wang et al. [12] and Li et al. [62]) were analyzed by RT-qPCR in the WT, deletion mutants, and complementary strains collected from Czapek plates at 5 dpi. For analyses expression of melanin biosynthesis genes during microsclerotium formation, the cultures of WT and other mutants were prepared on BMM medium and samples were collected at different stages of microsclerotium development (at 48, 72, 120, 168, and 336 hpi).

Samples were collected on BMM medium with 60 or 120 μg/ml tricyclazole for the detection of genes expressed in processes such as cell aging, DNA repair, and melanin biosynthesis by RT-qPCR. After growing on Czapek and MM medium for 5 days, the WT, Δ*VdPKS1*, Δ*VdPKS9*, and Δ*VdPKS9_1* strains were collected and used to determine gene expression levels in the intrinsic and the scytalone-exposed conditions. The expression of *VdPKS9* in the two hyphal types (AH and MH) of strains was examined in the BMM conditions (2–3.5 dpi) after growing on Czapek plates for 2 days, while AT13 and Vd991 strains were induced under corresponding stress (starvation BMM, 4 °C low temperature, high pH 10.0, 0.5 mM H_2_O_2_ treatment, UV exposure 1 h) conditions for 1.5 days (2–3.5 dpi) and then cultured on Czapek for 1.5 days (3.5–5 dpi). Additionally, DNA was extracted from the cotton stems of plants used in the pathogenicity assay to detect the biomass of *V. dahliae*.

Total RNA for the expression analyses were carried out by liquid nitrogen grinding and extractions described above. The RT-qPCR was performed to analyze gene expression with the primer pairs listed in Additional file 1: Table S3. The *V. dahliae* elongation factor 1α gene *VdEF-1α* was used for normalization. In fungal biomass analysis, qPCR was carried out with the cotton 18S rDNA gene (*Gh18S*) and tobacco *NbEF* as internal reference genes to quantify DNA of *V. dahliae* using the target of *VdEF-1α*. The amplification reaction was carried out using 2 × Top Green qPCR SuperMix (TransGen Biotech, Beijing, China) and the QuantStudio3 Real-Time PCR system (Thermo Fisher Scientific, USA). The cycling parameters included pre-denaturation at 95 °C for 3 min, followed by 40 cycles of 95 °C denaturation for 15 s, 60 °C annealing for 20 s, and 72 °C extension for 20 s. The 2^−ΔΔCT^ method [95] based on CT values was used to calculate the relative expression ratios. To better present the results, some gene expression levels were converted to heat maps using HemI (Heatmap Illustrator, version 1.0). There were three replicates for the qPCR and RT-qPCR experiments, enabling calculations of mean and standard error.

### Comparative transcriptome analysis

The WT AT13 and Δ*VdPKS9* strains of *V. dahliae* were cultured in liquid CM medium for 3 days and conidia were collected and adjusted to a concentration to 1 × 10^8^/ml. The conidial suspensions were coated on a microfiltration membranes overlaid on Czapek medium and grown for 5 days. The total RNA was extracted by the method described above, and the concentration and quality of the extracted RNA were measured. There were three biological replicates for both the WT strain and the mutant strain. Illumina HiSeq XTen was used to construct and sequence the DNA library. To obtain clean reads, reads containing adapters and low-quality reads were removed from the raw data, and the GC content was calculated from the clean reads using the *V. dahliae* strain AT13 (DK185) genome as a reference.

For the RNAseq analysis, differentially expressed genes (DEGs, in Additional file 1: Table S4) from three biological replicates of the mutant and WT were identified by Deseq R with a false discovery rate of 1% (fold-change ≥ 2.0, *P* < 0.01). The DEGs were analyzed using Gene Ontology (GO) analysis in the GO-seq package and Kyoto Encyclopedia of Genes and Genomes (KEGG) analysis [96, 97] using the KEGG Orthology (KO)-Based Annotation System (KOBAS) to explore their biological roles based on the classification system including plasma membrane, cell periphery, regulation of cellular process, cellular component organization, regulation of biological process, oxidation-reduction process, oxidoreductase activity, and melanin biosynthesis. The threshold for the significant enrichment of GO terms was *P* <0.05.

### Extraction and quantification of melanin and intermediate products

The extraction of melanin and its intermediate followed the method of Ma et al. [98]. The mycelium and fermentation broth used to extract metabolites were collected from BMM plate and Czapek liquid medium respectively. All strains were grown for 2 weeks, and the hyphae were ground into powder after drying. The powder was immersed in a solution that contained 0.2 M NaOH and an equal volume of acetone, and shaken for 10 min. After incubating at room temperature for 24 h, acetone was removed by rotary evaporation, and the remaining alkali solutions were adjusted to pH 2 with HCl and extracted with an equal volume of ethyl acetate three times. For the culture filtrates, the filtrate solutions were acidified and extracted with ethyl acetate in a similar way. The organic phase was concentrated, dried, and dissolved with ethyl acetate for TLC (thin layer chromatography) analysis, which was performed with the developing solvents of petroleum ether-ethyl acetate-formic acid (2:1:0.01 v/v/v), followed by chromogenic reaction with 5% vanillin-sulfuric acid.

The intermediate scytalone was obtained from the Δ*bcscd1* strain of *B. cinerea* (donated by Prof. Pin-Kuan Zhu of East China Normal University) using a similar method described by Chen et al. [32]. The Δ*bcscd1* mutant was grown in YSS liquid medium for 3 weeks, and with the broth adjusted to pH 2.0 with HCl. The extraction was performed three times with ethyl acetate containing 1% formic acid and concentrated by rotary evaporation. The target chemical was purified by column chromatography and eluted with petroleum ether and ethyl acetate (2:1 v/v). The molecular weight and content of scytalone were identified by nuclear magnetic resonance spectrometry (Bruker 500) and high-resolution mass spectrometry (Bruker MicroTOF-Q II LC MS) according to the instructions for the instruments (MS condition: Capillary (kV): 2.20; Ion Source: ESI+; Cone: 12v; Source Temperature (°C): 150; Desolvation Temperature (°C): 500; Cone Gas Flow (L/Hr): 150; Desolvation Gas Flow (L/Hr): 1000. LC condition: Injection volume: 5 μl; flow rate: 0.3 ml/min; mobile phase: A: 0.1% formic acid water; B: acetonitrile; Chromatographic column: ACQUITY UPLC BEH C18 1.7 μm 2.1 × 50 mm Column).

### Statistical analysis

In all experiments, the mean ± SD was calculated for each treatment with at least three replicates. Significant differences among treatments were identified using one-way analysis of variance (ANOVA) followed by least significant difference tests. Statistical analyses were performed using the SPSS version 19 software package (SPSS Inc., Chicago, IL, USA).

## Supplementary Information

**Additional file 1: Table S1**. Functional and PKS_domain information of VdPKSs in *Verticillium dahliae*. T**able S2**. Information on the primer pairs used to construct the vector in this study. **Table S3**. Information of RT-qPCR primer pairs used in this study. **Table S4**. Information of differentially expressed genes (DEGs) for comparative transcriptome analysis. **Table S5**. Information of strains in this study.

**Additional file 2: Figure S1**. Pathogenicity of 27 *VdPKSs*-deleted mutants in cotton. **Figure S2**. Relative fungal biomass of 27 *VdPKSs*-deleted mutants in cotton. **Figure S3**. Multi-sequence alignment of VdPKS9 and filamentous fungal quinone oxidoreductase PIG3 family homologues. **Figure S4**. Sensitivity of WT, Δ*VdPKS9* and EC^Δ*VdPKS9*^ strains to oxidation, osmotic stress, and cell wall inhibitors. **Figure S5**. Analyses of penetration of WT, Δ*VdPKS9* and EC^Δ*VdPKS9*^ strains on cellophane membranes. **Figure S6**. Microsclerotia production in the WT, Δ*VdPKS9* and EC^Δ*VdPKS9*^ strains. **Figure S7**. Melanin deposition phenotype of WT, Δ*VdPKS1*, Δ*VdPKS9* and Δ*VdPKS9_1* strains exposed to 50μg ml^-1^ scytalone. **Figure S8**. Colony morphology of WT, Δ*VdPKS1* and Δ*VdPKS1*-OE^*VdPKS9*^ strains. **Figure S9**. Colony morphology of and *B. cinerea*, *C. gloeosporioides* and *C. fructicola* and the relative expression level of *VdPKS9* homologues. **Figure S10**. Characteristics of hypha-type and microsclerotium-type strains. **Figure S11**. Response of *VdPKS9* to autophagy, programmed cell death and DNA damage. **Figure S12**. Colony diameter, scytalone content and melanin intermediate characteristics.

**Additional file 3.** Raw data of experimental results.

## Data Availability

All data generated or analyzed during this study are included in the article, its supplementary information files are publicly available in repositories. The RNA-seq data presented in this study have been deposited in the NCBI Sequence Read Archive (SRA) database under project accession number PRJNA835225 [99].
