# Supplementary material for: A polyketide synthase from Verticillium dahliae modulates melanin biosynthesis and hyphal growth to promote virulence
Source: BMC Biol. 2022 May 30;20:125. doi: 10.1186/s12915-022-01330-2 (PMC9153097; doi:10.1186/s12915-022-01330-2)
Supplement: Supplementary file 2 — Additional file 2: Figure S1. Pathogenicity of 27 VdPKSs-deleted mutants in cotton. Figure S2. Relative fungal biomass of 27 VdPKSs-deleted mutants in cotton. Figure S3. Multi-sequence alignment of VdPKS9 and filamentous fungal quinone oxidoreductase PIG3 family homologues. Figure S4. Sensitivity of WT, ΔVdPKS9 and ECΔVdPKS9 strains to oxidation, osmotic stress, and cell wall inhibitors. Figure S5. Analyses of penetration of WT, ΔVdPKS9 and ECΔVdPKS9 strains on cellophane membranes. Figure S6. Microsclerotia production in the WT, ΔVdPKS9 and ECΔVdPKS9 strains. Figure S7. Melanin deposition phenotype of WT, ΔVdPKS1, ΔVdPKS9 and ΔVdPKS9_1 strains exposed to 50μg ml-1 scytalone. Figure S8. Colony morphology of WT, ΔVdPKS1 and ΔVdPKS1-OEVdPKS9 strains. Figure S9. Colony morphology of and B. cinerea, C. gloeosporioides and C. fructicola and the relative expression level of VdPKS9 homologues. Figure S10. Characteristics of hypha-type and microsclerotium-type strains. Figure S11. Response of VdPKS9 to autophagy, programmed cell death and DNA damage. Figure S12. Colony diameter, scytalone content and melanin intermediate characteristics. [file 12915_2022_1330_MOESM2_ESM.docx]

# Supplementary Figures

## Figure S1


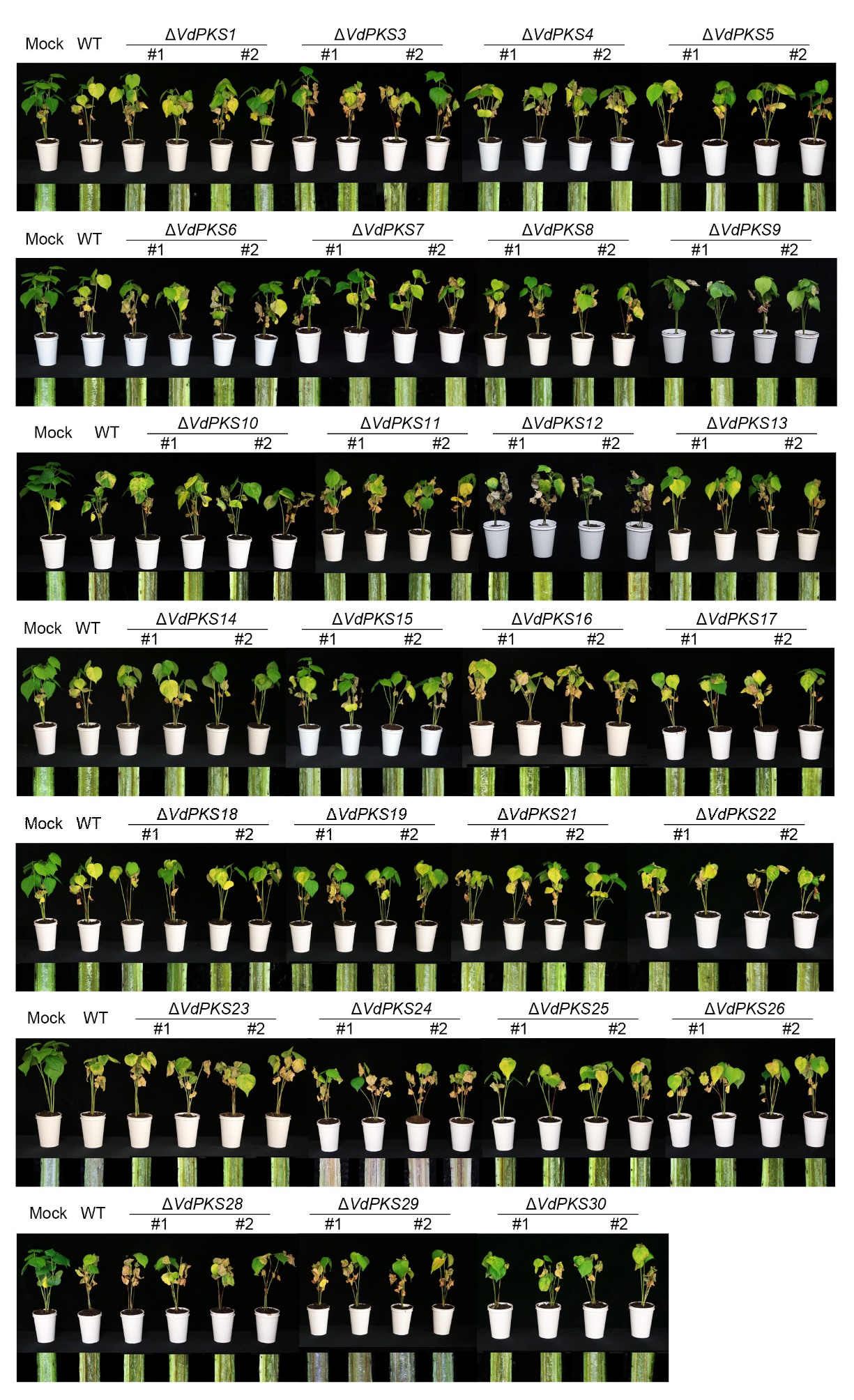


**Figure S1. Pathogenicity of 27 *VdPKSs*-deletion mutants of *Verticillium dahliae* in cotton.** Two single knockout transformants of each *VdPKS* were randomly selected and incubated in CM liquid medium for 3 days. The conidia were centrifuged, resuspended in sterile water, and diluted to a concentration of 5×10^6^ ml^-1^. Four-week-old cotton plants were inoculated with the conidia suspensions with WT (AT13) as control. The Verticillium wilt phenotype were examined at 21 dpi and longitudinal sections of cotton for root discoloration analyses. The pathogenicity of each respective *VdPKS* was analyzed with three replicates of 20 cotton plants.

## Figure S2


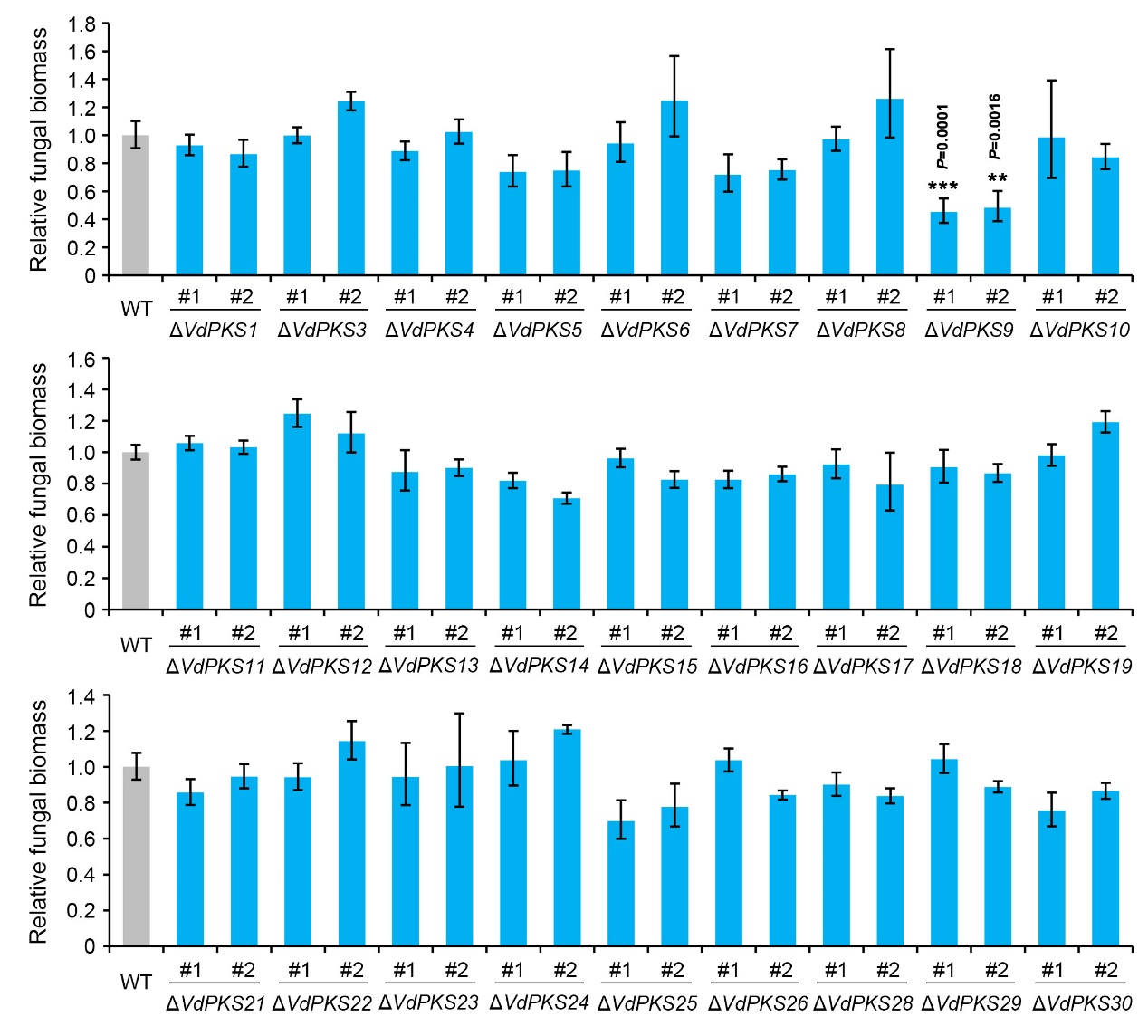


**Figure S2. Relative fungal biomass of 27 *VdPKSs*-deletion mutants of *Verticillium dahliae* in cotton.** Cotton stem bases were collected at 21 dpi. Samples were ground into powder for extraction of total DNA which was adjusted to similar concentration. Quantitative PCR analysis was conducted with WT as control. The cotton 18S rDNA gene (*Gh18S*) was used as internal reference to detect *VdEF-1α* expression of *V. dahliae* in each sample. The expression ratio was calculated by three independent biological replicates from cycle threshold (CT) values using the 2^-ΔΔCT^ method. The error bars represent SD (standard deviations), and asterisks indicate a significant difference (one-way ANOVA; **, *P* < 0.01; ***, *P* < 0.001).

## Figure S3


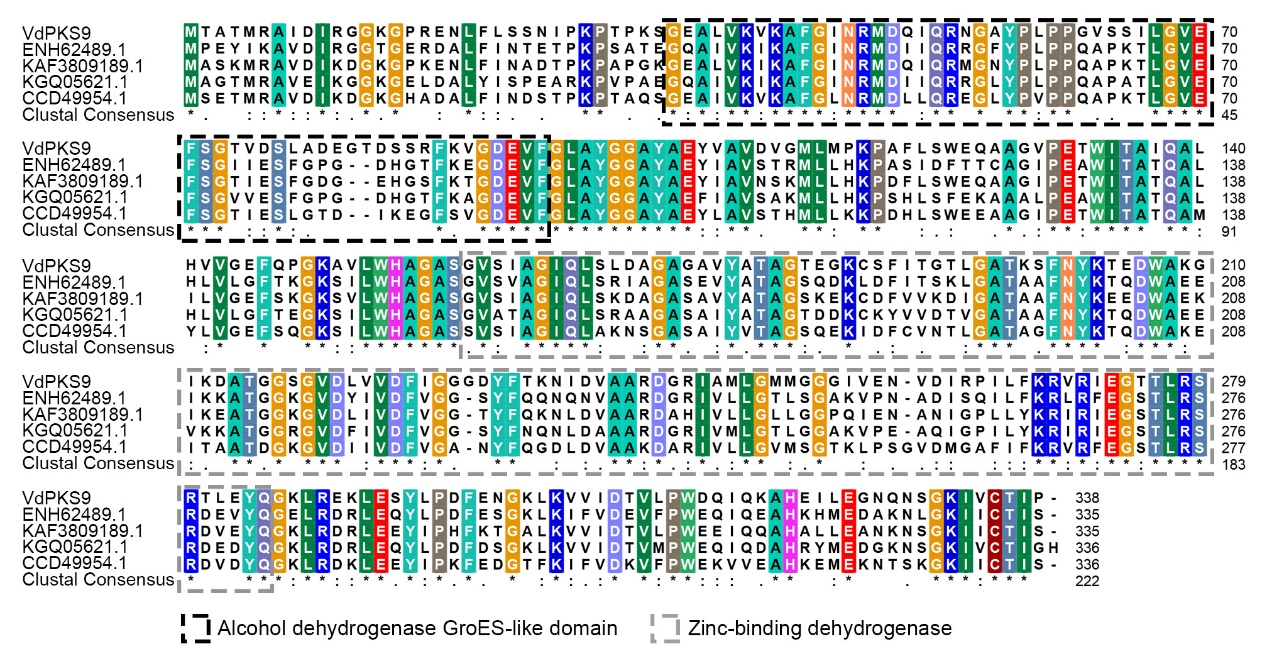


**Figure S3. Multi-sequence alignment of VdPKS9 from Verticillium dahliae with those of quinone oxidoreductase PIG3 family homologues from filamentous fungi.** Candidates were obtained by comparing VdPKS9 using the NCBI BLASTp tool. Multi-sequence alignment was performed by ClustalW. The black and gray dashed boxes divide the alcohol dehydrogenase GroES-like domain and zinc-binding dehydrogenase domain. (ENH62489.1 *Fusarium oxysporum* f. sp. cubense race 1; KAF3809189.1 *Colletotrichum gloeosporioides*; KGQ05621.1 *Beauveria bassiana* D1-5; CCD49954.1 *Botrytis cinerea* T4).

## Figure S4


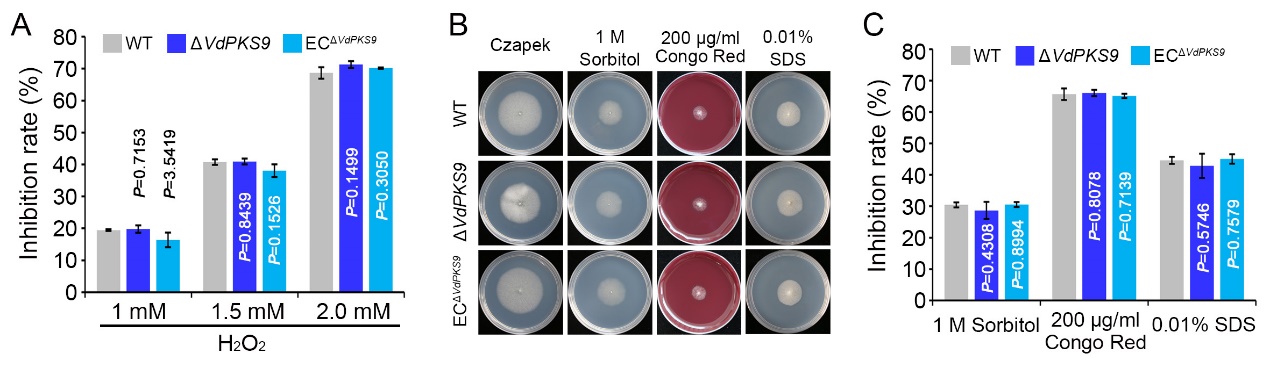


**Figure S4. Sensitivity of WT, Δ*VdPKS9* and EC^Δ^*^VdPKS9^* strains of *Verticillium dahliae* to oxidation, osmotic stress, and cell wall inhibitors. (A) and (C).** Sensitivity of WT, Δ*VdPKS9* and EC^Δ^*^VdPKS9^* strains to different stresses. These results show the growth inhibition rates (average colony diameters) on each plate compared with the control at 7 dpi. **(B).** Colony morphology of WT, Δ*VdPKS9* and EC^Δ^*^VdPKS9^* strains on medium containing oxidation, osmotic stresses and cell wall inhibitors. Photographs of the strains were obtained after cultivation on Czapek medium and Czapek with 1M sorbitol, 200 μg/ml Congo red, and 0.01% (W/V) SDS at 25°C for 7 days. Error bars represent SD of at least three independent repeated measurements, and significant differences depend on *P*-value (one-way ANOVA, SPSS).

## Figure S5


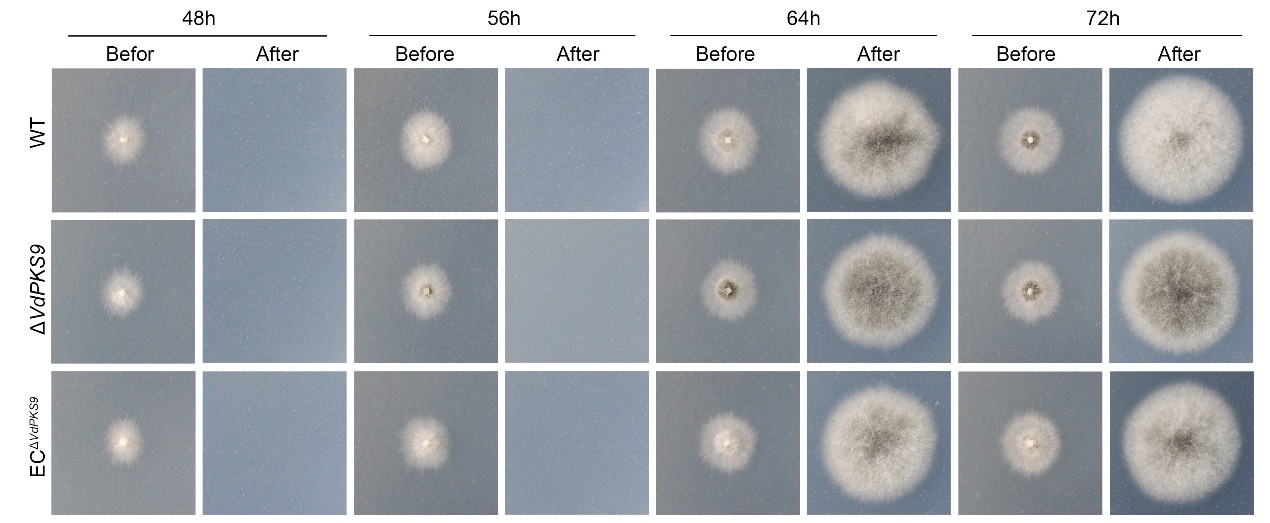


**Figure S5. Analyses of penetration of WT, Δ*VdPKS9* and EC^Δ^*^VdPKS9^* strains of *Verticillium dahliae* on cellophane membranes.** The hyphal blocks were obtained from MM plates, and placed on cellophane membranes for 48h. The cellophane membranes were removed every 8 hours for analyses. After treatment, plates were incubated at 25°C in the dark up to 8 dpi.

## Figure S6


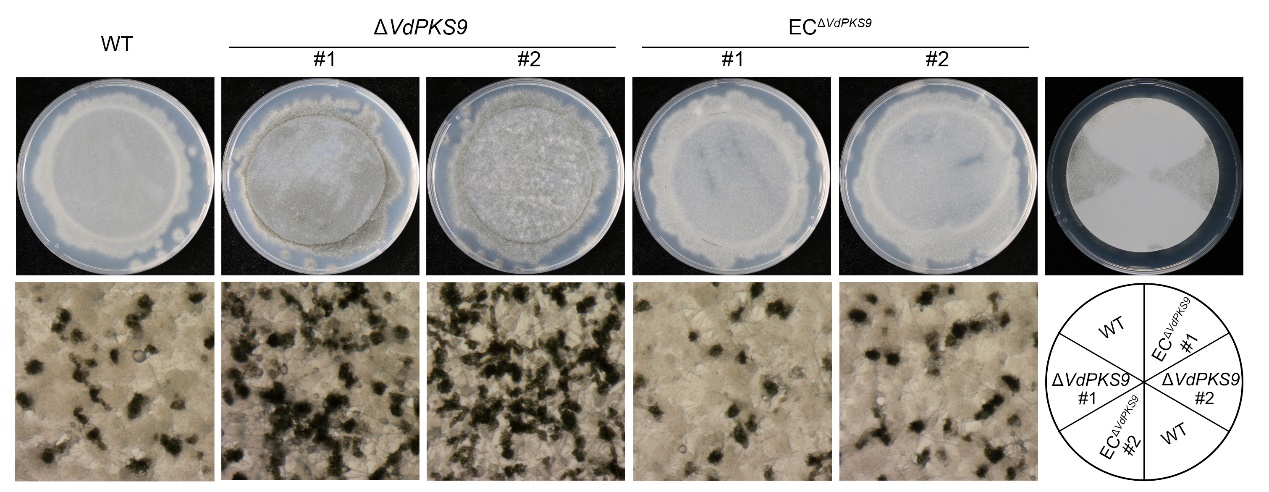


**Figure S6. Microsclerotia production in WT, Δ*VdPKS9* and EC^Δ^*^VdPKS9^* strains of *Verticillium dahliae*.** The conidia of different strains were harvested from PDA plates and 60 or 10 μl of a conidial suspension (1 × 10^6^/ml) was smeared onto a cellophane membrane or 1/6 of a microfiltration membrane covered on BMM medium. The morphology of microsclerotia was observed under a stereomicroscope after 7 days of incubation at 25 °C in dark.

## Figure S7


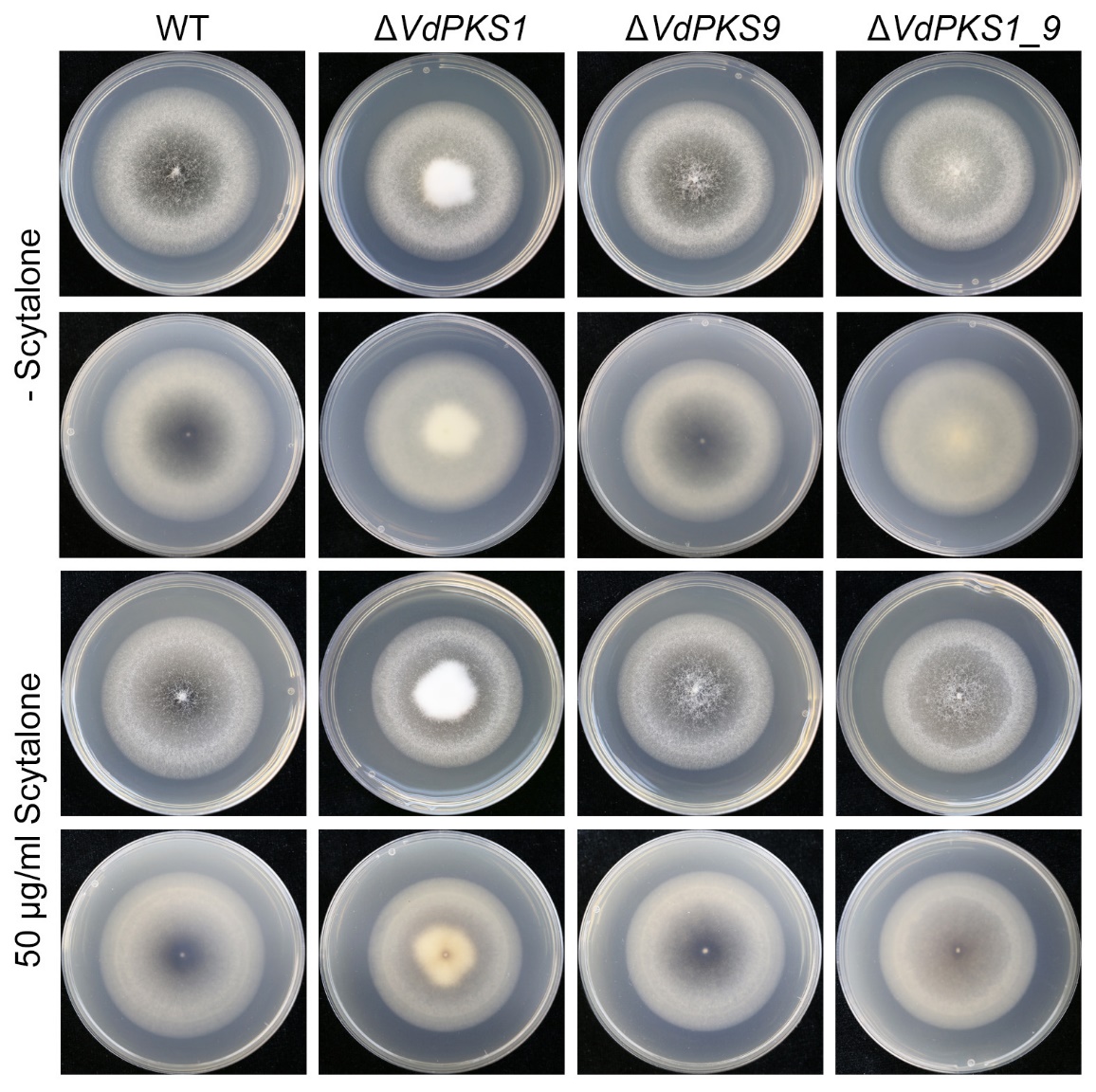


**Figure S7. Melanin deposition phenotype of WT, Δ*VdPKS1*, Δ*VdPKS9* and Δ*VdPKS9_1* strains of *Verticillium dahliae* exposed to 50 μg ml^-1^ scytalone.** The photographs show the colony morphology of each strain inoculated on MM plates containing scytalone for 10 days. MM plates containing equal volume of 10% ethanol were used as control, and all strains were repeated at least 3 plates.

## Figure S8


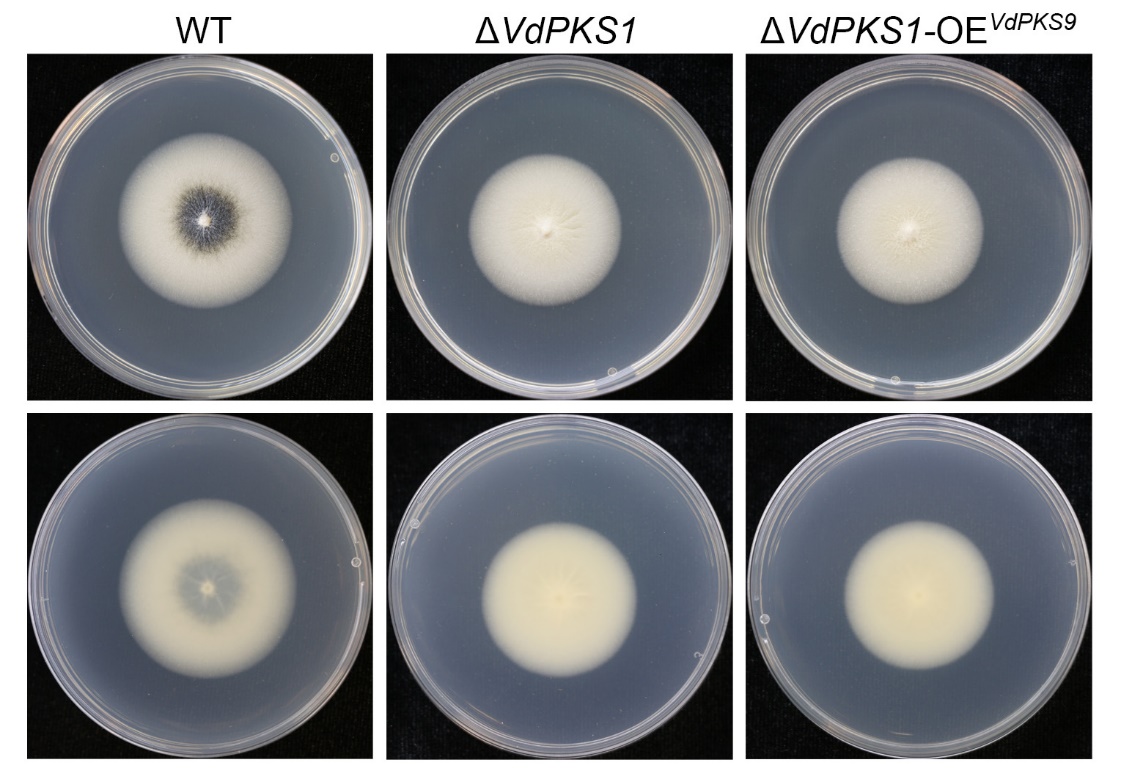


**Figure S8. Colony morphology of WT, Δ*VdPKS1* and Δ*VdPKS1*-OE*^VdPKS9^* strains of *Verticillium dahliae*.** The photographs show melanin deposition and hyphal growth phenotypes of WT, ΔVdPKS1 and ΔVdPKS1-OE*^VdPKS9^* strains on PDA medium for 7 days.

## Figure S9


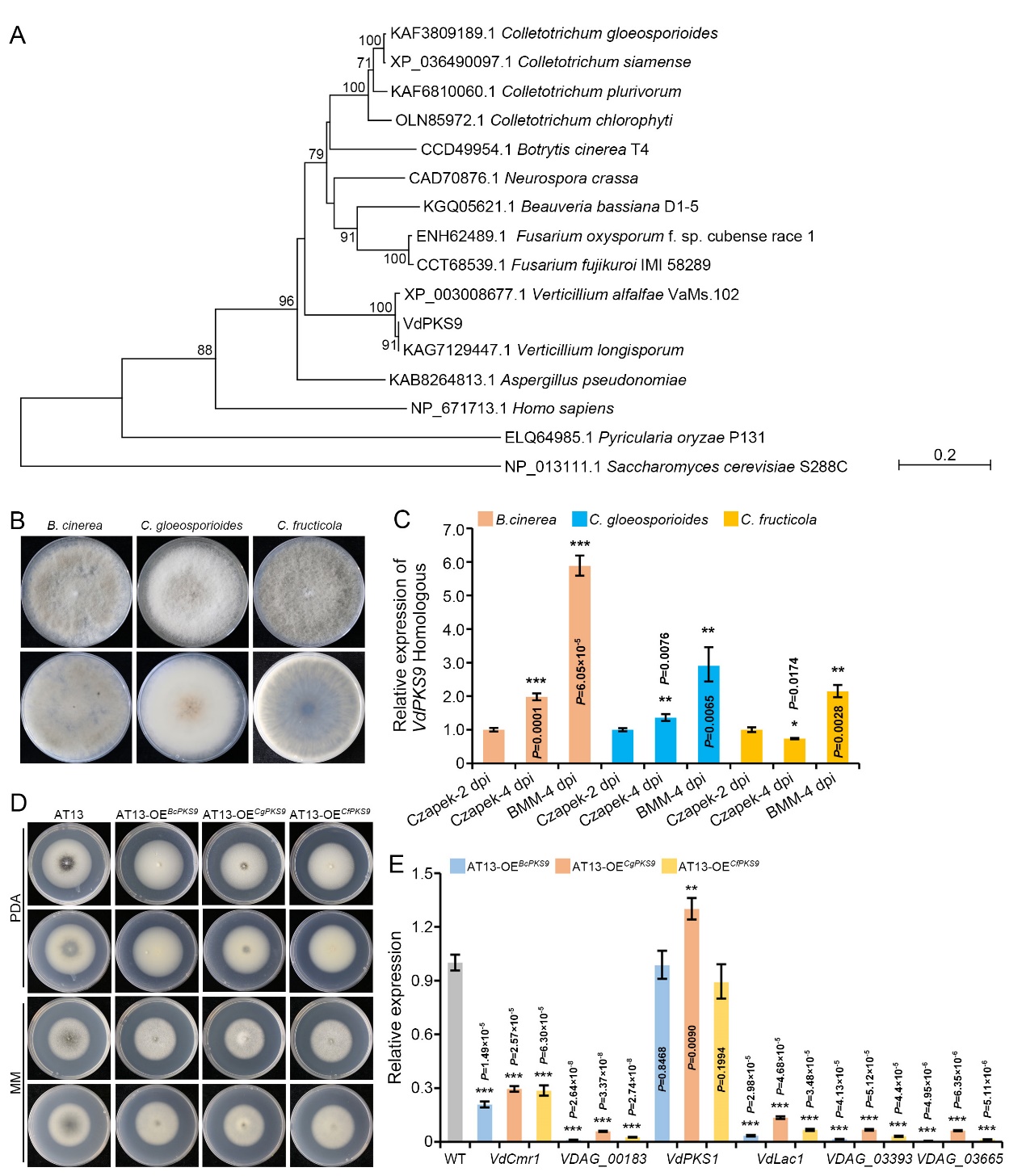


**Figure S9. Colony morphology of** ***B. cinerea*, *C. gloeosporioides* and *C. fructicola* and the relative expression level of *VdPKS9* homologues. (A).** Phylogenetic analysis of *VdPKS9* in *Verticillium dahliae* and its homologues from 15 other species. **(B).** Culture characteristics of three melanin producing fungi (*B. cinerea*, *C. gloeosporioides* and *C. fructicola*) grown on PDA plate for 7 days. **(C).** The relative gene expression ratio of *VdPKS9* homologues in *B. cinerea*, *C. gloeosporioides* and *C. fructicola*. The samples for RT-qPCR analysis were collected and RNA was extracted after growing on Czapek medium for 2 days, then the strains were transferred to Czapek and BMM for additional 2 days, respectively. The *VdEF-1α* of *V. dahliae* was used as internal reference to detect homologues expression with 2 dpi as control. **(D).** The colony morphology of heterologous expressed strains. The VdPKS9 homologues were cloned from *B. cinerea*, *C. gloeosporioides* and *C.* *fructicola*, and then overexpressed into WT. The differences of melanin enrichment between mutants and WT were observed on the PDA and MM medium grown for 7 days. **(E).** The relative expression of melanin biosynthesis genes in the PKS9 heterologously expressed strains compared with WT. Samples were collected from Czapek medium at 5 dpi. The expression ratio was calculated by three independent biological replicates from cycle threshold (CT) values using the 2^-ΔΔCT^ method. The error bars represent SD (standard deviations), and asterisks indicate a significant difference (one-way ANOVA; **, *P* < 0.01; ***, *P* < 0.001).

## Figure S10


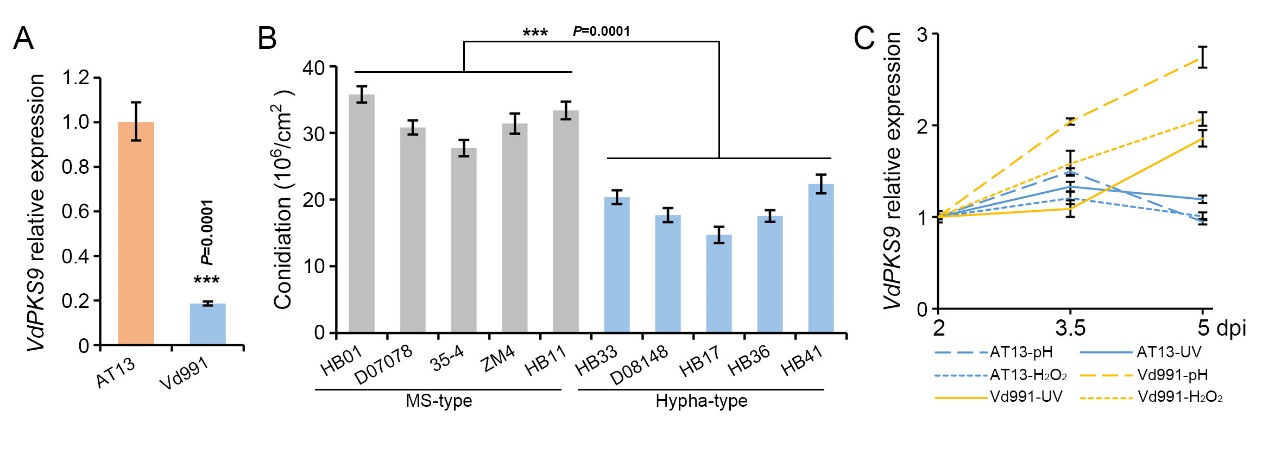


**Figure S10. Characteristics of hypha-type and microsclerotium-type strains of *Verticillium dahliae*. (A).** The relative expression of *VdPKS9* in *V. dahliae* AT13 and Vd991 strain examined by reverse transcription-quantitative PCR. The samples for gene quantitative analysis were collected and RNA was extracted after growing on Czapek medium for 2 days. **(B).** The conidiation of different hypha-type and microsclerotium-type strains. Strains grew on PDA plates for 7 days, and 3 hyphal plugs (diameter= 0.5 cm) were shaken in sterile water containing 0.05% Tween-20 for 1 minute before counted by hemocytometer. **(C).** The relative expression of *VdPKS9* in *V. dahliae* AT13 and Vd991 strain after abiotic stress treatment. The samples for gene quantitative analysis were collected and RNA was extracted after growing in different environments (Czapek medium for 2 days; after growing on Czapek medium with pH=10 or 0.8 mM H_2_O_2_ for 1.5 days, strains turned to normal culture for another 1.5 days; 1 hour UV exposure and recovery for 1.5 and 3 days). The relative expression of *VdPKS9* at was used as control. The *VdEF-1α* of *V. dahliae* was used as internal reference for normalization in the expression studies. The expression ratio was calculated by three independent biological replicates from cycle threshold (CT) values using the 2^-ΔΔCT^ method. The error bars represent SD (standard deviations), and asterisks indicate a significant difference (one-way ANOVA; **, *P* < 0.01; ***, *P* < 0.001).

## Figure S11


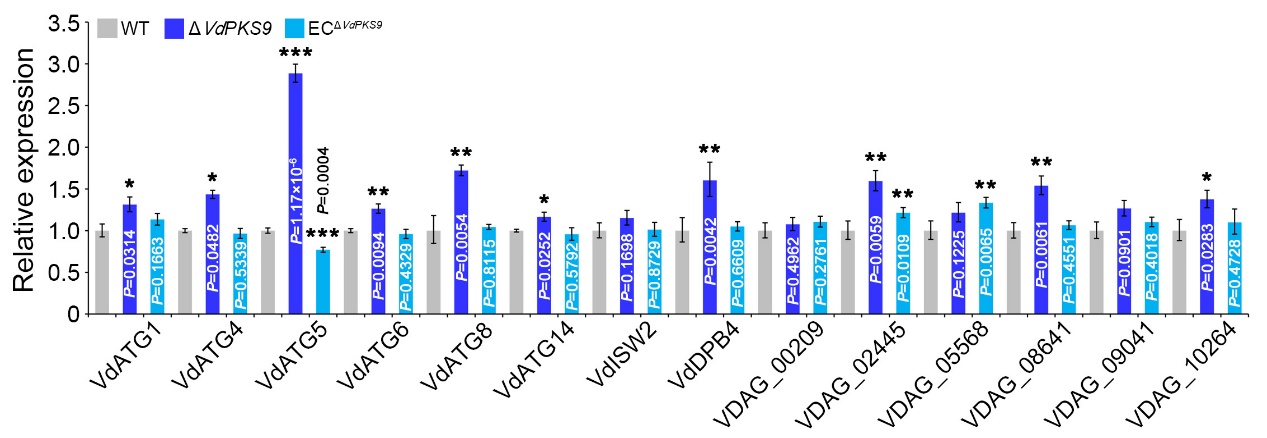


**Figure S11. Relative expression analyses of target genes associated with autophagy, programmed cell death and DNA damage in a *Verticillium dahliae*** *VdPKS9* **mutant background.** Samples were collected from BMM medium at 5 dpi and RNA was extracted. The reverse transcribed cDNA was used for analyses of the expression of target genes, which were homologues of autophagy genes of *M. oryzae* and known DNA repair-related genes of *V. dahliae*. The *VdEF-1α* of *V. dahliae* was used as internal reference to detect target gene expression by reverse transcription-quantitative PCR. The expression ratio was calculated by three independent biological replicates from cycle threshold (CT) values using the 2^-ΔΔCT^ method. The error bars represent SD (standard deviations), and asterisks indicate a significant difference (one-way ANOVA; **, *P* < 0.01; ***, *P* < 0.001).

## Figure S12


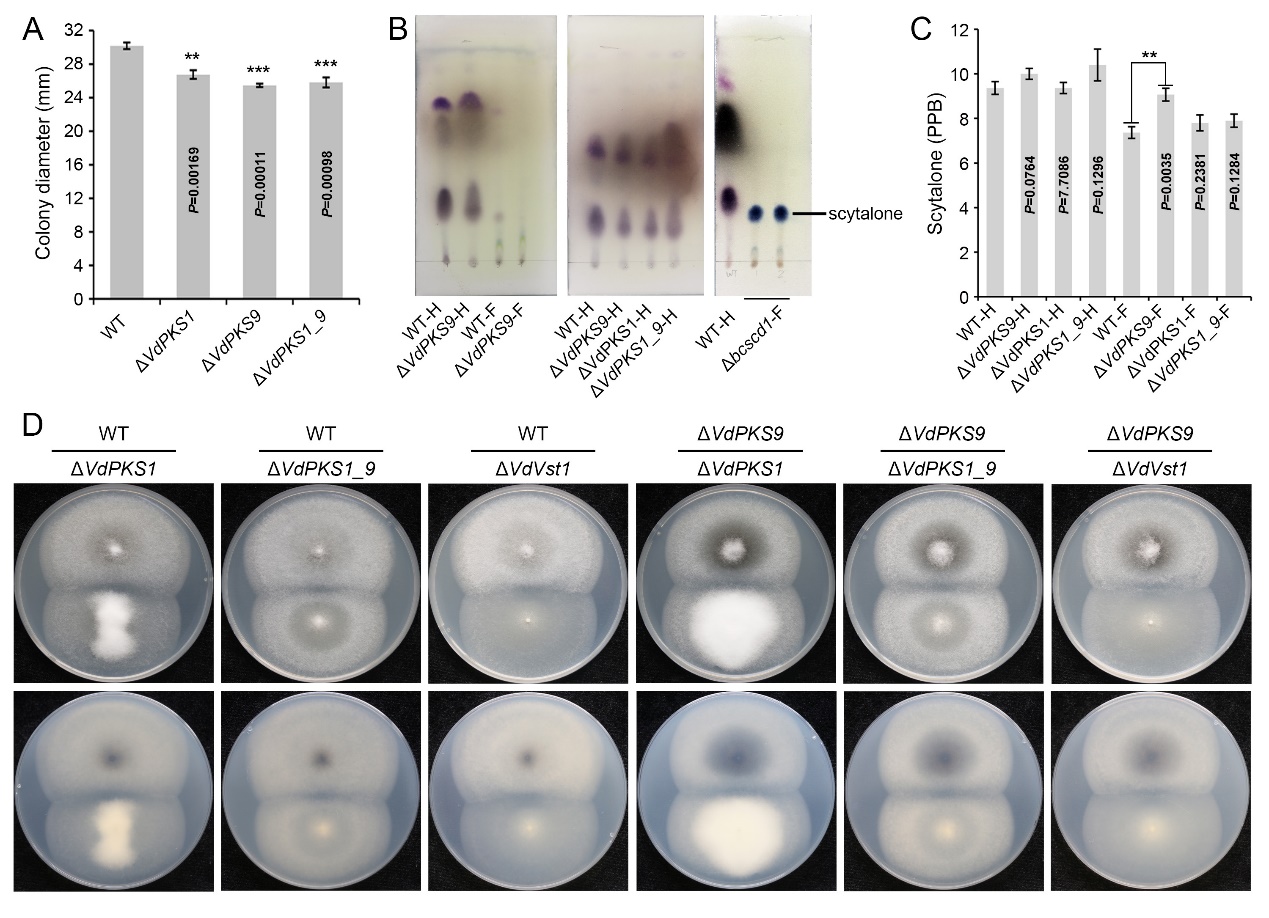


**Figure S12. Colony diameter, scytalone content and melanin intermediate characteristics in strains of *Verticillium dahliae*. (A).** Colony diameter of WT, Δ*VdPKS1*, Δ*VdPKS9* and Δ*VdPKS9_1* mutants. Each strain grew on PDA plates at 25 °C for 7 days, and at least three colony diameters were measured. **(B).** Detection of secondary metabolites in fermentation broth (-F) and hyphae (-H) of WT, Δ*VdPKS1*, Δ*VdPKS9* and Δ*VdPKS9_1* mutants by TLC. The mixed compounds of each strain were obtained according to the melanin extraction method. Then, TLC was performed with the developing solvents and chromogenic reaction with vanillin-sulfuric acid. Preliminary purified scytalone derived from mutants was used as control. **(C).** The purified scytalone was used as the standard, and its content in different samples was quantitatively detected by UPLC-MS/MS. **(D).** Culture phenotypes of WT and Δ*VdPKS9* against different hyaline strains. The melanin accumulation in hyaline strains was observed after the two strains grew on MM plate for 14 days. All experiments were repeated 3 times, error bars represent SD of at least 3 independent repeated measurements, and asterisks indicate a significant difference (one-way ANOVA; **, *P* < 0.01; ***, *P* < 0.001).
